# Energy Use and Economic Performance Nexus in Sub-Saharan Africa: A Multivariate Analysis

**DOI:** 10.12688/openresafrica.15788.1

**Published:** 2025-05-13

**Authors:** Mfonobong Effiong, Manoj Panicker

**Affiliations:** 1 Department of Economics and Business Sciences, Walter Sisulu University, Mthatha, Eastern Cape, 5117, South Africa; 2 The Executive Deans Office, Faculty of Economic and Financial Sciences, Walter Sisulu University, Mthatha, Eastern Cape, South Africa

**Keywords:** Energy use, Natural resources, low-carbon energy output, Multivariate analysis, Sub-Saharan Africa

## Abstract

**Background:**

Sub-Saharan Africa's energy landscape is complex, with various factors influencing economic growth and development. Understanding the interplay between energy use, economic performance, and natural resources is crucial for sustainable development. This study investigates the relationships between energy use, GDP, low-carbon energy output, natural resources, and economic performance in Sub-Saharan Africa.

**Methods:**

This study employed advanced econometric techniques, including generalized linear models, generalized method of moments, and vector error correction models. Data from the Global Economy Database spanning 1990-2024 were analyzed to uncover relationships between energy use, GDP, low-carbon energy output, and natural resources.

**Results:**

The analysis showed significant variations in low-carbon energy output (mean = 68.86 units), natural gas profit margin (mean = $0.10), and oil operating surplus (mean = $3.50). The GLM and GMM estimates revealed significant relationships between energy use and GDP (7.419%), low-carbon energy output (6.079%), natural gas profit margin (67.377%), and oil operating surplus (4.575%). The analysis revealed significant variability in low-carbon energy production, natural gas profitability, and oil operating surpluses. Statistical models showed strong correlations between energy consumption and GDP growth, as well as low-carbon energy output and natural resource utilization.

**Conclusions:**

The study finds complex dynamics between the variables, with both short-term and long-term effects. The research contributes to existing knowledge by providing empirical evidence of the relationships between energy use, GDP, low-carbon energy output, natural resources, and economic performance, offering valuable insights for policymakers and stakeholders seeking to promote sustainable energy use and economic development in Sub-Saharan Africa. This research provides novel insights into the intricate relationships governing Sub-Saharan Africa's energy sector and economic development. The findings offer valuable guidance for policymakers and stakeholders seeking to promote sustainable energy use, economic growth, and environmental stewardship in the region.

## Introduction

The relationship between energy use, economic performance, and natural resources has been a subject of interest for decades, with numerous studies exploring the dynamics between these variables ( Yu-Ke *et al.*, 2022). The increasing energy demand and concerns about climate change and sustainable development have led to a growing body of research on understanding the complex interactions between energy use, economic growth, and natural resources ( Fatima *et al.*, 2025). The link between energy use and economic performance has been extensively studied, with many researchers finding a positive relationship between energy consumption and economic growth ( Gahlot & Garg, 2024). For instance, Apergis and Payne (2010) found that energy consumption and economic growth are cointegrated, suggesting a long-run relationship between the two variables. Similarly, the role of natural resources in economic development has also been a topic of interest, with some researchers arguing that abundant natural resources can lead to economic growth ( Amare *et al.*, 2024). However, others have found that the relationship between natural resources and economic growth is more complex, with factors such as institutional quality and human capital playing a crucial role ( Faisal *et al.*, 2025).

However, the increasing concern about climate change has led to a growing interest in low-carbon energy sources, such as renewable energy ( Pearce *et al.*, 2014). Most studies rely on traditional econometric methods, which may not capture the complex dynamics between variables with particular emphasis on developed economies, neglecting the experiences of developing countries. Studies have shown that transitioning to low-carbon energy sources can have numerous benefits, including reducing greenhouse gas emissions and improving energy security ( Edenhofer *et al.*, 2011). Despite the extensive research on energy use, economic performance, and natural resources, there is still a need for studies that explore the complex relationships between these variables in a comprehensive and integrated manner. This study aims to fill this research gap by investigating the relationships between energy use, economic performance, and natural resources, focusing on low-carbon energy output and resource-based revenue.

## Methods

Sub-Saharan Africa is a vast region encompassing 46 countries with diverse climate zones, geography, and ecosystems. The region's climate is influenced by its latitude, longitude, and altitude, resulting in varying temperature and rainfall patterns. It experiences a range of rainfall patterns, from arid deserts to tropical rainforests. The region's rainfall is primarily determined by its latitude, with the equatorial regions receiving more rainfall than the subtropical regions, and the temperature varies greatly, with average temperatures ranging from 18°C to 30°C ( Zita *et al.*, 2025). Temperature is influenced by altitude, latitude, and longitude. Based on the geographic coordinates, its latitude and longitude span between 20°N and 35°S and 20°W to 50°E, respectively. Sub-Saharan Africa can be divided into several climate regions, including the Mediterranean (characterized by mild winters and hot summers), Sahara (hot and arid desert region), Western Africa (tropical savanna climate), and Eastern Africa (with diverse climate, ranging from tropical to temperate) ( Rouault *et al.*, 2024).

### Data sources

Secondary data used for the study were sourced from the global economy database, for the period, 1990–2023 forecasted to 2024 (using ARIMA techniques) in the forty-six (46) countries that makes up Sub-Saharan Africa, comprising Angola, Benin, Botswana, Burkina Faso, Burundi, Cameroon, Cape Verde, Central African Republic, Chad, Comoros, Côte d'Ivoire, the Democratic Republic of the Congo, Djibouti, Equatorial Guinea, Eritrea, Eswatini (formerly Swaziland), Ethiopia, Gabon, Gambia, Ghana, Guinea, Guinea-Bissau, Kenya, Lesotho, Liberia, Madagascar, Malawi, Mali, Mauritania, Mauritius, Mozambique, Namibia, Niger, Nigeria, Republic of the Congo, Rwanda, São Tomé and Principe, Senegal, Seychelles, Sierra Leone, Somalia, South Africa, South Sudan, Sudan, Tanzania, and Togo.

### Research design

The study utilizes a quantitative research design to analyze the relationship between energy use and economic growth in Sub-Saharan Africa. A combination of dynamic panel data analysis techniques, including GMM, GLM, VECM, and VAR models, was employed to ensure robust and accurate findings.

### Variable description and measurement

Each variable used in the study is operationalized as follows:

**Dependent Variable:** Energy use (EU) (kWh per capita).

**Explanatory Variables:**

GDP = Gross domestic product, measured in constant 2010 (USD).

CEO = Low-carbon energy output (% of total energy).

RBR = Resource-based revenue (% of GDP).

NGPM = Natural gas profit margin (%).

OOS = Oil operating surplus (%). These variables are included to assess their direct and indirect impacts on energy use and greenhouse gas emissions.

### Data cleaning and preprocessing

The data obtained from the global economy database underwent preprocessing steps, including:

❖ **Outlier Removal:** Identifying and handling extreme values to avoid bias.
❖ **Handling Missing Data:** Employing interpolation methods to fill gaps in the dataset.
❖ **Standardization:** Ensuring the comparability of variables across countries and years through normalization or unit conversion.

### Justification for methodology

The selection of dynamic panel data estimation methods, such as GMM and VECM, is justified by the following:

❖ **Lagged Effects:** Capturing the influence of past values on current observations.
❖ **Endogeneity Control:** Addressing potential endogeneity between variables.
❖ **Cross-country comparisons:** Evaluating the relationships across 46 Sub-Saharan African countries.

### Model validation

The validity of the models was assessed using diagnostic tests, including:

❖ **Sargan/Hansen Test:** Checking the validity of instrumental variables in the GMM model.
❖ **Serial Correlation Test:** Ensuring no second-order autocorrelation in the error terms.
❖ **Stationarity Test:** Confirming the stationarity of variables using unit root tests.

### Ethical considerations

The study strongly emphasizes ethical guidelines by using publicly available secondary data, ensuring that no privacy or confidentiality issues arise. The sources are appropriately acknowledged, maintaining transparency and academic integrity. This commitment to ethical considerations reassures the audience about the integrity of our research.

### Model specification

**Generalized linear models (GLM):**


$$EU=\beta_{0}+\beta_{1}GDP+\beta_{2}CEO+\beta_{3}RBR+\beta_{4}NGPM+\beta_{5}OOS+\varepsilon$$

**Generalized Method of Moments (GMM) model:**


$$Y_{it}=\beta_{0}+\beta_{1}GDP_{it}+\beta_{2}CEO_{it}+\beta_{3}RBR_{it}+\beta_{4}NGPM_{it}+\beta_{5}OOS_{it}+\varepsilon_{it}$$

**Vector error correction model (VECM):**


$$\begin{array}{l} \Delta EU=(\propto EU_{-1}-\beta_{0}-\beta_{1}GDP_{-1}-\beta_{2}CEO_{-1}-\beta_{3}RBR_{-1}-\beta_{4}NGPM_{-1}-\beta_{5}OOS_{-1})+Y_{1}\Delta GDP\\ \;+Y_{2}\Delta CEO+Y_{3}\Delta RBR+Y_{4}\Delta NPRM+Y_{5}\Delta OOS+\varepsilon \end{array}$$

**Cointegrating Equation:**


$$EU=\beta_{0}+\beta_{1}GDP+\beta_{2}CEO+\beta_{3}RBR+\beta_{4}NGPM+\beta_{5}00S+\varepsilon$$

**Vector autoregression (VAR) equation:**


$$EU=\beta_{0}+\beta_{1}EU_{-1}+\beta_{2}GDP_{-1}+\beta_{3}RBR_{-1}+\beta_{4}NPRM_{-1}+\beta_{5}OOS_{-1}+\beta_{6}CEO_{-1}+\varepsilon$$

$$GDP=\beta_{0}+\beta_{1}EU_{-1}+\beta_{2}GDP_{-1}+\beta_{3}RBR_{-1}+\beta_{4}NPRM_{-1}+\beta_{5}OOS_{-1}+\beta_{6}CEO_{-1}+\varepsilon$$

$$RBR=\beta_{0}+\beta_{1}EU_{-1}+\beta_{2}GDP_{-1}+\beta_{3}RBR_{-1}+\beta_{4}NPRM_{-1}+\beta_{5}OOS_{-1}+\beta_{6}CEO_{-1}+\varepsilon$$

$$NPRM=\beta_{0}+\beta_{1}EU_{-1}+\beta_{2}GDP_{-1}+\beta_{3}RBR_{-1}+\beta_{4}NPRM_{-1}+\beta_{5}OOS_{-1}+\beta_{6}CEO_{-1}+\varepsilon$$

$$OOS=\beta_{0}+\beta_{1}EU_{-1}+\beta_{2}GDP_{-1}+\beta_{3}RBR_{-1}+\beta_{4}NPRM_{-1}+\beta_{5}OOS_{-1}+\beta_{6}CEO_{-1}+\varepsilon$$

$$CEO=\beta_{0}+\beta_{1}EU_{-1}+\beta_{2}GDP_{-1}+\beta_{3}RBR_{-1}+\beta_{4}NPRM_{-1}+\beta_{5}OOS_{-1}+\beta_{6}CEO_{-1}+\varepsilon$$

Where:

*Y _it_ = Energy use*

*β*_1_, *β*_2_, *β*_3_, *β*_4_, *and* *β*_5_ = Parameters to be estimated

*GDP _it_ = Gross domestic product at time (t)*

*CEO _it_ = low – carbon energy output at time (t)*

*RBR _it_ = resource – based revenue at time (t)*

*NGPM _it_ = Natural gas profit margin at time (t)*

*OOS _it_ = Oil operating surplus at time (t)*

*ε _it_ = error term at time (t)*

## Results and discussion

### Socioeconomic characteristics of the variables

The descriptive statistics for six variables related to energy, economy, and natural resources are presented in Table 1.

**Table 1.** Descriptive statistics.

	Energy use	GDP	Low-carbon energy output	Natural gas profit margin	Oil operating surplus	Resource- based revenue
Mean	624.0407	7.898686	68.85657	0.102623	3.498742	11.41208
Median	624.0407	7.898686	74.89500	0.000000	0.000000	9.445000
Maximum	3103.970	198.6300	98.34000	6.630000	82.78000	88.59000
Minimum	9.580000	0.650000	0.710000	0.000000	0.000000	0.000000
Std. Dev.	344.1328	7.959100	23.80336	0.445301	9.126025	10.44448
Skewness	4.116855	17.52244	-1.175478	7.968621	3.607843	2.090501
Kurtosis	24.56398	379.9210	3.646439	78.31642	17.19267	9.195289
Jarque-Bera	36496.76	9815885.	407.2241	405969.3	17364.62	3826.573
Probability	0.000000	0.000000	0.000000	0.000000	0.000000	0.000000
Sum	1025923.	12985.44	113200.2	168.7121	5751.932	18761.47
Sum Sq. Dev.	1.95E+08	104079.6	930923.7	325.7947	136836.2	179230.1
Observations	1644	1644	1644	1644	1644	1644

Results showed that the mean energy use per capita and low-carbon energy out were approximately 624 and 68.86 units, respectively, with a standard deviation of 344 and 23.80 units, indicating significant (moderate) variations in energy consumption across different regions or countries. On the other hand, GDP and natural resources resulting from natural gas profit margin and oil operating surplus had a mean of $7,898, $0.10, and $3.50, respectively, with a standard deviation of $7,959, $0.45, and $9.13 suggesting substantial disparities in economic performance, variations (low) in profit margins in the natural gas and oil sectors. The mean resource-based revenue also had a similar result of $11.41, with a standard deviation of $10.44, suggesting moderate variations in the income generated from natural resources.

The skewness values indicate that the distributions of energy use, GDP, natural gas profit margin, and oil operating surplus were positively skewed, while low-carbon energy output was negatively skewed. The kurtosis values suggest that the distributions of the variables were leptokurtic, indicating a higher concentration of extreme values. These results suggest rejecting the null hypothesis of normality and non-normal distribution for the variables, as indicated by the Jarque-Bera standard statistical normality test.

These findings are consistent with recent research by Wang (2011) on Decoupling measure between economic growth and energy consumption of China who found significant variations in energy consumption across different regions. Similarly, the positive skewness in energy use, GDP, natural gas profit margin, and oil operating surplus were in line with Fattouh and Mahadeva (2014), which found that oil prices and revenues can exhibit asymmetric distributions.

**Figure 1.**
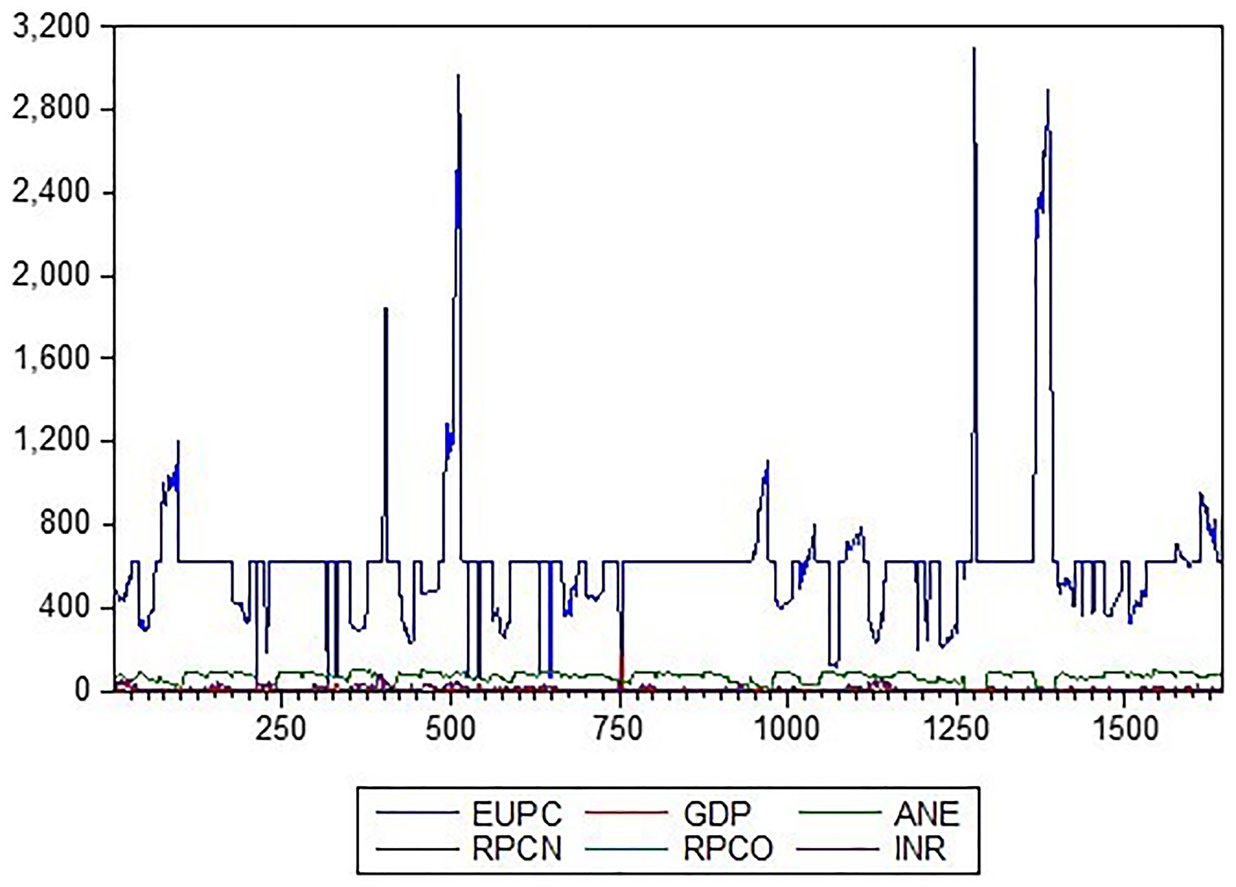
Line plot description of the variables. *KEY: EUPC=Energy use, ANE=low-carbon energy output, INR=resource-based revenue, RPCN=natural gas profit margin, RPCO=oil operating surplus, GDP=gross domestic product*.

As presented in Figure 1, the variables exhibit distinct patterns. The energy use per capita plot steadily increases over time, with some fluctuations indicating growing energy demands. In contrast, the low-carbon energy output plot shows a more erratic pattern, with occasional spikes, reflecting the variable nature of alternative energy sources. The GDP plot exhibits an upward trend, with some oscillations indicating economic growth. The Natural Gas Profit Margin and Oil Operating Surplus plots display more volatile patterns, with frequent fluctuations reflecting changes in global energy markets. Finally, the Resource-Based Revenue (RBR) plot showed a steady increase, with some variations, indicating growing revenue from natural resources. These graphical representations provide valuable insights into the relationships and trends among these descriptive variables.

### Exploring linear relationships and predicting outcomes

Generalized linear models (GLMs) offer a flexible framework for analyzing complex relationships between variables, extending traditional linear regression to accommodate diverse data types and distributions. By leveraging GLMs, researchers can model and predict outcomes in various fields, uncovering patterns and trends that inform decision-making and policy development. GLMs provide a robust toolset for understanding the relationships between variables, accounting for non-normal responses and non-constant variance, and enabling the exploration of nuanced interactions and effects.

As presented in Table 2, the relationships between the variables showed that low-carbon energy output, resource-based revenue, oil operating surplus, natural gas profit margin, and GDP had highly significant coefficients (p-values < 0.01), indicating a strong relationship with energy use. The coefficient values explain that a 1% increase in GDP, low-carbon energy output, natural gas profit margin, and oil operating surplus is associated with a 7.419%, 6.079%, 67.377%, and 4.575% increase in energy use. At the same time, resource-based revenue had a relatively small effect with a significant coefficient of 0.0314. This implies that the variables showed strong relationships with energy use due to their significant coefficients, indicating that increases in GDP, low-carbon energy output, natural gas profit margin, and oil operating surplus are associated with substantial increases in energy consumption. At the same time, resource-based revenue has a relatively minor effect. The log-likelihood coefficient (-12308.80) indicated a reasonable model fit, with an Akaike Information Criterion (AIC) value of 14.98030 and a deviance value (3.07E+08), indicating moderate model complexity and a reasonable model fit. The AIC and deviance values provide insights into the magnitude of the relationships between the variables and the overall model performance.

**Table 2.** Generalized linear model (Quadratic Hill Climbing).

Variable	Coefficient	Std. Error	z-Statistic	Prob.
GDP	7.419610	1.239101	5.987896	0.0000 ***
Resource-based revenue	3.523273	1.637452	2.151680	0.0314 **
Low-carbon output	6.079725	0.271558	22.38832	0.0000 ***
Natural gas profit margin	67.37751	24.58739	2.740327	0.0061 ***
Oil operating surplus	4.575330	1.871061	2.445313	0.0145 **
Mean dependent var	624.0407	S.D. dependent var		344.1328
Sum squared resid	3.07E+08	Log-likelihood		-12308.80
Akaike info criterion	14.98030	Schwarz criterion		14.99673
Hannan-Quinn criteria.	14.98639	Deviance		3.07E+08
Deviance statistic	187096.1	Pearson SSR		3.07E+08
Pearson statistic	187096.1	Dispersion		187096.1

*Sample/observations:11644, Dispersion computed using Pearson Chi-Square, Coefficient covariance computed using Hessian, Convergence achieved after 1 iteration*.

### Non-linear relationships (Outcome variables)

Generalized method moments (GMM) models have been known to handle non-linear relationships between the predictors and the response variable. This provides insights into the relationships between the dependent variable (energy use) and the independent variables as presented.

Table 3 shows that GDP, low-carbon energy output, natural gas profit margin, and oil operating surplus had significant coefficients at a 5% level, indicating a strong relationship with energy use. The coefficient values suggest that a 1% increase in GDP, low-carbon energy output, natural gas profit, and oil operating surplus in Africa is associated with 13.405%, 5.977%, 56.694%, and 10.289% increase in energy use with resource-based output having an insignificant and no-strong relationship with energy use. This implies that the variables significantly affect energy use and provides insights into the magnitude of the relationships between the variables.

**Table 3.** Generalized method of moments.

Variable	Coefficient	Std. Error	t-Statistic	Prob.
Resource-based revenue	-1.615789	2.203357	-0.733331	0.4635
GDP	13.40532	6.452842	2.077429	0.0379**
Low-carbon output	5.977299	0.650924	9.182795	0.0000***
Natural gas profit margin	56.69458	24.96224	2.271214	0.0233**
Oil Operating surplus	10.28933	4.421084	2.327333	0.0201**
S.E. of regression	437.2268	Mean dependent var		624.0407
Durbin-Watson stat	1.86818	S.D. dependent var		344.1328
Instrument rank	7	Prob(J-statistic)		0.000071
Sum squared resid J-statistics	3.13E+08 19.09173			

**Figure 2.**
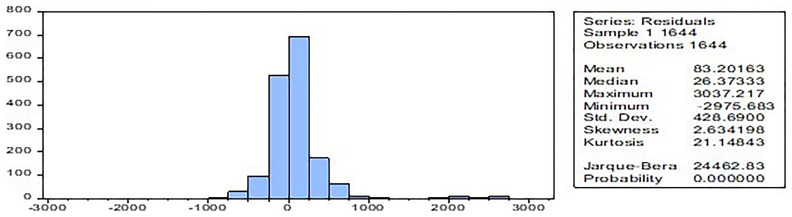
Normality test.

The normality test, Figure 2 has confirmed that the model's residuals follow a normal distribution, which is significant at a 1% level (Jarque-Bera test and skewness statistics).

**Figure 3.**
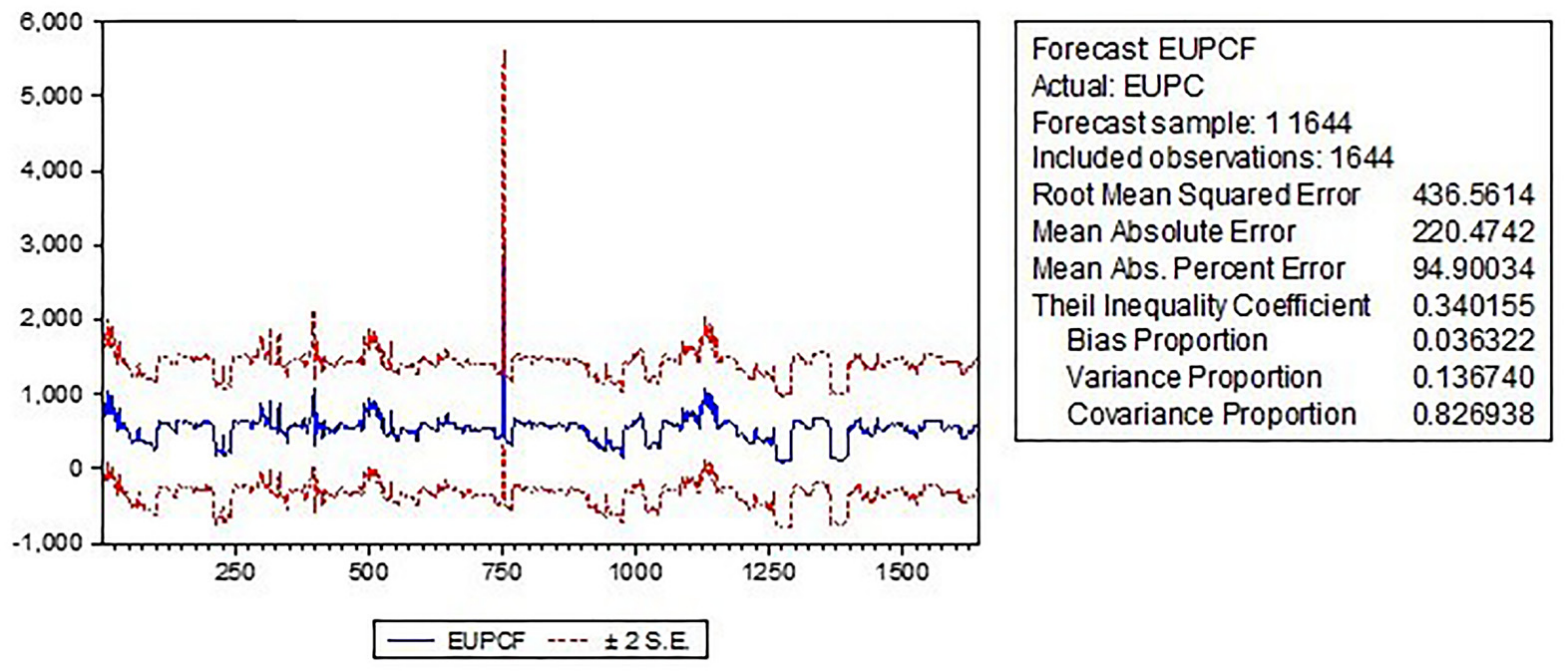
Forecast graphical presentation.

The forecast graph Figure 3 visually represents the predicted values of the dependent variable (energy use) over a specified forecast horizon. It shows that energy use is expected to increase over the forecast horizon with some fluctuations, and the actual values of energy use are generally within the confidence intervals, indicating that the model has reasonable predictive power.

### Long-run and short-run dynamics

**Figure 4.**
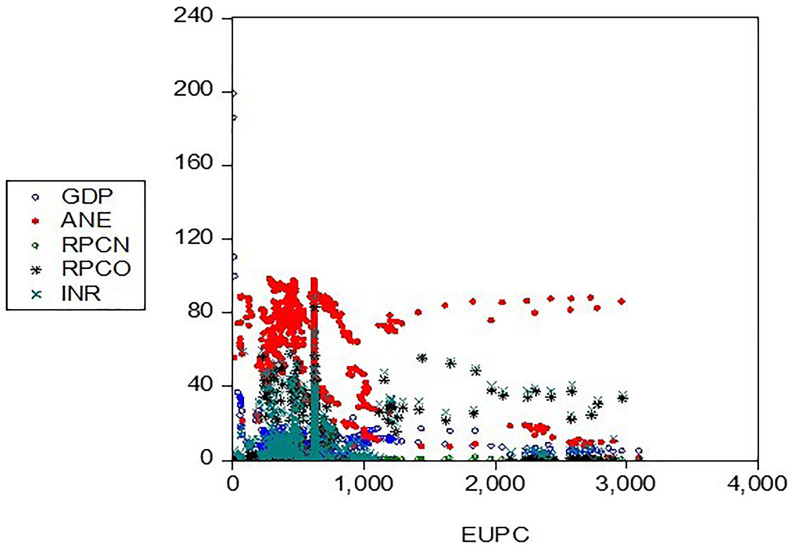
Unit root (stationarity) of the variables. *KEY: EUPC=Energy use, ANE=low-carbon energy output, INR=resource-based revenue, RPCN=natural gas profit margin, RPCO=oil operating surplus, GDP=gross domestic product*.

The unit root test was carried out to confirm the stationarity of the variables as presented:

Table 4 presents the group unit root test results, which suggests the rejection of the null (H _0_) hypothesis of a unit root ( Figure 4). Levin, Lin & Chu t*, Im, Pesaran, and Shin W-stat, ADF - Fisher Chi-square, and PP - Fisher Chi-square test assumed a standard unit root process across all variables (test statistic =-66.4144, and -58.1983) significant at a 1% level, rejecting the null hypothesis of a unit root.

**Table 4.** Unit root test.

Method	Statistic	Prob. **	Cross-sections	Obs
Null: Unit root (assumes standard unit root process)				
Levin, Lin & Chu t*	-66.4144	0.0000***	6	9809
Null: Unit root (assumes individual unit root process)				
Im, Pesaran and Shin W-stat	-58.1983	0.0000***	6	9809
ADF - Fisher Chi-square	930.052	0.0000***	6	9809
PP - Fisher Chi-square	136.957	0.0000***	6	9852

** Probabilities for Fisher tests are computed using an asymptotic Chi-square distribution. All other tests assume asymptotic normality, automatic lag length selection based on SIC: 0–15, Newey-West automatic bandwidth selection, and Bartlett kernel.

As presented in Table 5, the VECM estimates provide insights into the variables' short-term dynamics and long-term relationships. Based on the findings, the cointegrating equation (CointEq1) indicates a long-run relationship among the variables, with energy use, GDP, low-carbon energy output, and oil operating surplus having significant coefficients. The error correction terms (ECT) represent the short-term adjustments of the variables toward their long-run equilibrium. The ECTs are substantial for most variables, indicating that the system adjusts to restore equilibrium. The estimates of the short-term dynamics suggest that the variables exhibit significant short-term relationships, with some variables having a more substantial impact on others.

**Table 5.** Vector Error Correction Estimates.

Cointegrating Eq:	CointEq1					
EUPC(-1)	1.000000					
GDP(-1)	593.9147					
	(36.3323)					
	[16.3468]					
ANE(-1)	31.93588					
	(11.2977)					
	[2.82676]					
INR(-1)	29.86489					
	(40.1244)					
	[0.74431]					
RPCN(-1)	738.3781					
	(601.562)					
	[1.22744]					
RPCO(-1)	-66.25970					
	(45.1468)					
	[-1.46765]					
C	-7700.986					
Error Correction:	D(EUPC)	D(GDP)	D(ANE)	D(INR)	D(RPCN)	D(RPCO)
CointEq1	0.003248	-0.000570	-3.53E-05	2.13E-05	-8.07E-07	1.54E-05
	(0.00090)	(3.6E-05)	(4.0E-05)	(3.2E-05)	(1.3E-06)	(2.5E-05)
	[3.59053]	[-16.0216]	[-0.88556]	[0.66918]	[-0.60975]	[0.61295]
D(EUPC(-1))	-0.042425	0.000848	-0.000353	0.001125	-6.37E-06	0.001518
	(0.02544)	(0.00100)	(0.00112)	(0.00089)	(3.7E-05)	(0.00070)
	[-1.66766]	[0.84824]	[-0.31434]	[1.25796]	[-0.17106]	[2.15473]
D(EUPC(-2))	-0.066936	-0.002245	-0.000431	-0.000625	1.28E-05	-0.000290
	(0.02544)	(0.00100)	(0.00112)	(0.00089)	(3.7E-05)	(0.00070)
	[-2.63124]	[-2.24479]	[-0.38381]	[-0.69917]	[0.34487]	[-0.41172]
D(GDP(-1))	-0.413579	0.096821	0.014877	0.023184	2.09E-05	0.018025
	(0.66357)	(0.02609)	(0.02928)	(0.02332)	(0.00097)	(0.01838)
	[-0.62327]	[3.71106]	[0.50806]	[0.99429]	[0.02153]	[0.98069]
D(GDP(-2))	-0.234531	0.007920	0.007334	-0.006509	0.000206	-0.005664
	(0.64859)	(0.02550)	(0.02862)	(0.02279)	(0.00095)	(0.01796)
	[-0.36160]	[0.31058]	[0.25623]	[-0.28560]	[0.21680]	[-0.31530]
D(ANE(-1))	-0.919150	0.010476	0.009899	0.037265	-0.001214	-0.004334
	(0.57163)	(0.02248)	(0.02523)	(0.02009)	(0.00084)	(0.01583)
	[-1.60795]	[0.46613]	[0.39240]	[1.85526]	[-1.45112]	[-0.27370]
D(ANE(-2))	-0.518210	-0.002727	0.007921	0.020278	-0.000269	-0.001132
	(0.57324)	(0.02254)	(0.02530)	(0.02014)	(0.00084)	(0.01588)
	[-0.90400]	[-0.12098]	[0.31311]	[1.00671]	[-0.32051]	[-0.07129]
D(INR(-1))	1.129644	0.006877	0.045484	-0.179934	-0.000147	0.037945
	(1.00342)	(0.03945)	(0.04428)	(0.03526)	(0.00147)	(0.02779)
	[1.12579]	[0.17430]	[1.02719]	[-5.10323]	[-0.10023]	[1.36525]
D(INR(-2))	0.084003	0.006617	0.012407	-0.115765	-0.000141	0.031313
	(0.99654)	(0.03918)	(0.04398)	(0.03502)	(0.00146)	(0.02760)
	[0.08429]	[0.16888]	[0.28213]	[-3.30596]	[-0.09656]	[1.13441]
D(RPCN(-1))	-21.49170	0.027722	2.490451	-0.531809	-0.374297	-0.613321
	(17.2675)	(0.67892)	(0.76200)	(0.60676)	(0.02528)	(0.47828)
	[-1.24463]	[0.04083]	[3.26832]	[-0.87648]	[-14.8066]	[-1.28234]
D(RPCN(-2))	-16.95231	-0.180831	0.262123	-0.445365	-0.071837	-0.473753
	(17.3269)	(0.68125)	(0.76462)	(0.60884)	(0.02537)	(0.47993)
	[-0.97838]	[-0.26544]	[0.34282]	[-0.73149]	[-2.83202]	[-0.98713]
D(RPCO(-1))	0.171729	-0.002034	-0.027041	-0.009631	0.001675	-0.214704
	(1.28049)	(0.05035)	(0.05651)	(0.04499)	(0.00187)	(0.03547)
	[0.13411]	[-0.04040]	[-0.47855]	[-0.21404]	[0.89365]	[-6.05353]
D(RPCO(-2))	1.875573	0.005375	-0.031866	-0.033140	0.002371	-0.156630
	(1.27270)	(0.05004)	(0.05616)	(0.04472)	(0.00186)	(0.03525)
	[1.47370]	[0.10743]	[-0.56739]	[-0.74103]	[1.27275]	[-4.44317]
C	0.031166	0.103305	-0.177476	-0.077785	0.003790	0.012361
	(3.88282)	(0.15266)	(0.17134)	(0.13644)	(0.00568)	(0.10755)
	[0.00803]	[0.67669]	[-1.03578]	[-0.57012]	[0.66673]	[0.11494]
Y1	-69.85736	-0.160243	5.569380	0.303755	-0.088899	-0.979442
	(21.4586)	(0.84370)	(0.94694)	(0.75403)	(0.03141)	(0.59437)
	[-3.25545]	[-0.18993]	[5.88142]	[0.40284]	[-2.82983]	[-1.64787]
Y2	63.01164	-1.401870	-0.004929	-2.046892	-0.027610	-1.008705
	(21.8102)	(0.85752)	(0.96246)	(0.76638)	(0.03193)	(0.60411)
	[2.88909]	[-1.63479]	[-0.00512]	[-2.67085]	[-0.86470]	[-1.66974]
Y3	0.743852	-0.690725	0.743655	-0.265215	-0.006901	-0.150428
	(21.9163)	(0.86170)	(0.96714)	(0.77011)	(0.03208)	(0.60705)
	[0.03394]	[-0.80159]	[0.76892]	[-0.34439]	[-0.21508]	[-0.24780]
Y4	9.322318	-0.391146	0.218646	-0.386938	0.002346	0.650019
	(21.3662)	(0.84006)	(0.94287)	(0.75078)	(0.03128)	(0.59181)
	[0.43631]	[-0.46561]	[0.23190]	[-0.51538]	[0.07501]	[1.09836]
Y5	-3.068481	-0.479627	-0.274619	2.387437	-0.003818	0.244234
	(21.2863)	(0.83692)	(0.93934)	(0.74797)	(0.03116)	(0.58960)
	[-0.14415]	[-0.57308]	[-0.29235]	[3.19188]	[-0.12252]	[0.41424]
Y6	3.268815	-0.492387	-0.015875	2.215856	-0.008064	0.127973
	(21.3860)	(0.84085)	(0.94374)	(0.75148)	(0.03131)	(0.59236)
	[0.15285]	[-0.58559]	[-0.01682]	[2.94867]	[-0.25756]	[0.21604]
R-squared	0.832767	0.162682	0.030020	0.063686	0.127674	0.051965
Adj. R-squared	0.811430	0.152868	0.018651	0.052712	0.117450	0.040853
Sum sq. resides	33211294	51340.28	64674.39	41006.81	71.17877	25479.91
S.E. equation	183.1368	5.627786	6.316473	5.029635	0.209548	3.964673
F-statistic	2.890272	16.57599	2.640437	5.803035	12.48690	4.676482
Log-likelihood	-10464.01	-5153.599	-5343.044	-4969.202	246.1413	-4578.769
Akaike AIC	12.77759	6.305422	6.536312	6.080685	-0.275614	5.604837
Schwarz SC	12.84344	6.371273	6.602163	6.146536	-0.209763	5.670688
Mean dependent	0.088063	0.000883	-0.002354	-0.010401	7.69E-06	-0.014340
S.D. dependent	144.6957	6.114515	6.376213	5.167678	0.223056	4.048227
Determinant resid covariance (of adj.)		1.96E+08				
Determinant resid covariance		1.82E+08				
Log-likelihood		-29577.99				
Akaike information criterion		36.20230				
Schwarz criterion		36.61716				

*KEY: EUPC=Energy use, ANE=low-carbon energy output, INR=resource-based revenue, RPCN=natural gas profit margin, RPCO=oil operating surplus, GDP=gross domestic product*

These results confirm the existence of a long-run relationship among the variables, consistent with the cointegration analysis. The error correction terms (significant) indicate that the system tends towards equilibrium, but the speed and dynamics of this adjustment can vary significantly across variables. Also, the short-term dynamics suggest that policymakers should consider the potential short-term effects of their actions on the variables.

These findings conform with the study by Parveen *et al.* (2020) who found a significant positive relationship between energy consumption and economic growth in Pakistan. Similarly, the significant error correction terms (ECTs) indicating short-term adjustments toward long-run equilibrium are consistent with research by Adhikari and Chen (2012).

### Impulse responses and dynamic relationships: unveiling the insights from vector autoregression estimates

Vector autoregression (VAR) estimates provide valuable insights into the dynamic relationships between multiple time series variables. By analyzing impulse responses, researchers can uncover the magnitude and direction of interactions between variables, shedding light on complex systems and feedback loops. VAR models capture the endogenous interactions between variables, allowing for a nuanced understanding of how shocks propagate through the system. They are a powerful tool for forecasting, policy analysis, and understanding interconnected economic, financial, or social phenomena.

As presented in Table 6, each variable is significantly affected by its lagged values and some other variables' lagged values. The relationships between variables are complex, with some positive and negative coefficients with a goodness of fit (R-squared = 0.84) varying across equations (most are above 0.7). The F-statistics and Log likelihood values indicate that all equations are significant. These findings provide more insight into the future values of the variables, thus simulating the effects of policy changes.

**Table 6.** Vector autoregression estimates.

	EUPC	GDP	ANE	INR	RPCN	RPCO
EUPC(-1)	0.919142	0.000315	-0.000710	0.000991	-7.03E-06	0.001451
	(0.02551)	(0.00102)	(0.00113)	(0.00090)	(3.7E-05)	(0.00071)
	[36.0260]	[0.30801]	[-0.62692]	[1.10204]	[-0.18842]	[2.03660]
EUPC(-2)	-0.009269	-0.001017	-5.41E-05	-0.001079	5.19E-06	-0.001431
	(0.02553)	(0.00102)	(0.00113)	(0.00090)	(3.7E-05)	(0.00071)
	[-0.36301]	[-0.99339]	[-0.04771]	[-1.19837]	[0.13898]	[-2.00641]
GDP(-1)	1.231940	0.758927	-0.006252	0.032530	-0.000384	0.024890
	(0.63278)	(0.02537)	(0.02809)	(0.02231)	(0.00093)	(0.01768)
	[1.94686]	[29.9176]	[-0.22255]	[1.45788]	[-0.41474]	[1.40817]
GDP(-2)	0.215885	-0.093281	-0.014308	-0.022947	-5.03E-06	-0.018037
	(0.63426)	(0.02543)	(0.02816)	(0.02237)	(0.00093)	(0.01772)
	[0.34037]	[-3.66868]	[-0.50812]	[-1.02598]	[-0.00542]	[-1.01810]
ANE(-1)	-0.997661	-0.005394	0.986229	0.035661	-0.001098	-0.005549
	(0.56735)	(0.02274)	(0.02519)	(0.02001)	(0.00083)	(0.01585)
	[-1.75846]	[-0.23717]	[39.1535]	[1.78250]	[-1.32357]	[-0.35016]
ANE(-2)	0.706866	-0.009260	-0.026151	-0.023232	0.000818	0.005109
	(0.56819)	(0.02278)	(0.02523)	(0.02004)	(0.00083)	(0.01587)
	[1.24407]	[-0.40655]	[-1.03668]	[-1.15952]	[0.98376]	[0.32193]
INR(-1)	1.180604	-0.010467	0.047440	0.774475	1.17E-05	0.034120
	(1.00848)	(0.04043)	(0.04477)	(0.03556)	(0.00148)	(0.02817)
	[1.17068]	[-0.25891]	[1.05956]	[21.7787]	[0.00792]	[1.21127]
INR(-2)	-0.896185	-0.006396	-0.040681	0.094085	-9.29E-05	-0.027753
	(1.00527)	(0.04030)	(0.04463)	(0.03545)	(0.00147)	(0.02808)
	[-0.89148]	[-0.15871]	[-0.91149]	[2.65415]	[-0.06319]	[-0.98838]
RPCN(-1)	-16.14026	-0.277202	2.738270	-0.275883	0.595486	-0.474104
	(16.5579)	(0.66378)	(0.73512)	(0.58387)	(0.02422)	(0.46250)
	[-0.97478]	[-0.41761]	[3.72491]	[-0.47251]	[24.5889]	[-1.02509]
RPCN(-2)	12.36142	-0.070517	-1.981541	0.118211	0.301922	0.086916
	(16.5328)	(0.66277)	(0.73401)	(0.58298)	(0.02418)	(0.46180)
	[0.74769]	[-0.10640]	[-2.69961]	[0.20277]	[12.4859]	[0.18821]
RPCO(-1)	-0.308487	0.028592	-0.031120	0.003417	0.001823	0.760409
	(1.27538)	(0.05113)	(0.05662)	(0.04497)	(0.00187)	(0.03562)
	[-0.24188]	[0.55922]	[-0.54959]	[0.07598]	[0.97741]	[21.3451]
RPCO(-2)	0.168184	0.002406	-0.000678	0.026106	0.000616	0.150589
	(1.27183)	(0.05099)	(0.05647)	(0.04485)	(0.00186)	(0.03553)
	[0.13224]	[0.04718]	[-0.01200]	[0.58212]	[0.33127]	[4.23895]
C	62.68981	4.314595	3.129913	0.468667	0.030968	0.265873
	(16.8371)	(0.67497)	(0.74752)	(0.59371)	(0.02463)	(0.47030)
	[3.72332]	[6.39228]	[4.18707]	[0.78938]	[1.25755]	[0.56533]
Y1	-70.05924	-0.162692	5.606720	0.293278	-0.089602	-0.991047
	(21.0666)	(0.84453)	(0.93530)	(0.74286)	(0.03081)	(0.58844)
	[-3.32560]	[-0.19264]	[5.99456]	[0.39480]	[-2.90801]	[-1.68419]
Y2	60.87124	-1.411254	0.147884	-2.011146	-0.031535	-1.028215
	(21.4178)	(0.85861)	(0.95089)	(0.75524)	(0.03133)	(0.59825)
	[2.84208]	[-1.64366]	[0.15552]	[-2.66292]	[-1.00666]	[-1.71870]
Y3	-1.243690	-0.587131	1.086892	0.030124	-0.011161	0.121689
	(20.9971)	(0.84174)	(0.93222)	(0.74041)	(0.03071)	(0.58650)
	[-0.05923]	[-0.69752]	[1.16592]	[0.04069]	[-0.36342]	[0.20748]
Y4	3.620679	-0.576980	0.455157	-0.372170	-0.003902	0.587410
	(20.9048)	(0.83804)	(0.92811)	(0.73715)	(0.03058)	(0.58392)
	[0.17320]	[-0.68849]	[0.49041]	[-0.50488]	[-0.12763]	[1.00598]
Y5	-2.140016	-0.481636	0.012973	2.096556	-0.008176	0.073779
	(20.9274)	(0.83894)	(0.92912)	(0.73795)	(0.03061)	(0.58455)
	[-0.10226]	[-0.57410]	[0.01396]	[2.84107]	[-0.26713]	[0.12621]
Y6	4.043505	-0.507520	0.251956	2.056337	-0.013670	-0.044457
	(21.0107)	(0.84229)	(0.93282)	(0.74089)	(0.03073)	(0.58688)
	[0.19245]	[-0.60255]	[0.27010]	[2.77551]	[-0.44483]	[-0.07575]
R-squared	0.835266	0.505141	0.932141	0.777222	0.789568	0.816219
Adj. R-squared	0.833439	0.499652	0.931389	0.774752	0.787234	0.814180
Sum sq. resides	32047829	51503.23	63170.02	39849.09	68.55734	25004.28
S.E. equation	140.5206	5.633236	6.238731	4.955072	0.205526	3.925075
F-statistic	457.1803	92.04000	1238.571	314.5718	338.3175	400.4524
Log-likelihood	-10440.61	-5158.841	-5326.477	-4948.218	277.5987	-4565.588
Akaike AIC	12.74009	6.306749	6.510934	6.050204	-0.314980	5.584152
Schwarz SC	12.80262	6.369276	6.573462	6.112731	-0.252453	5.646679
Mean dependent	624.1979	7.900189	68.85265	11.39732	0.102651	3.475653
S.D. dependent	344.3129	7.963832	23.81759	10.44045	0.445571	9.105464
Determinant resid covariance (of adj.)		1.68E+08				
Determinant resid covariance		1.57E+08				
Log-likelihood		-29470.57				
Akaike information criterion		36.03480				
Schwarz criterion		36.40997				

*KEY: EUPC=Energy use, ANE=low-carbon energy output, INR=resource-based revenue, RPCN=natural gas profit margin, RPCO=oil operating surplus, GDP=gross domestic product; Sample (adjusted): 31644, included observations: 1642 after adjustments, standard errors in ( ) $ t-stats in [ ]*

Consistent with recent studies, Apergis and Payne (2010) used a VAR model with cointegration to investigate the relationships between energy consumption, economic growth, and carbon emissions. Their findings emphasized the importance of considering the long-run relationships between these variables when assessing the impact of energy consumption on economic growth and environmental degradation. Bekiros and Paccagnini (2014) analyzed the relationships between GDP, inflation, and unemployment in the US using a VAR model with cointegration restrictions. Similar to this analysis, their findings highlighted the importance of accounting for long-run relationships when modeling economic time series. These studies showcase the versatility and growing applications of VAR models in various fields, including economics, neuroscience, and psychology.

**Figure 5.**
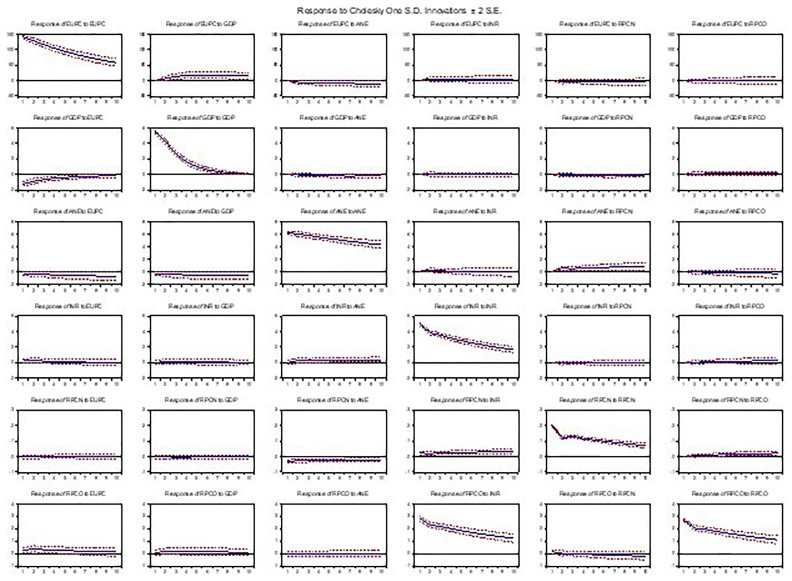
Impulse response analysis.

As presented, the graph in Figure 5 shows the dynamic responses of variables in a Vector Autoregression (VAR) model to shocks or impulses, providing insights into the short-term and long-term effects of shocks on the variables. It showed that a one-standard-deviation shock to energy use leads to a significant increase in GDP, low-carbon energy output, and oil operating surplus in the short term (first 2–3 periods), followed by a gradual decline. Resource-based revenue and natural gas profit margin exhibited a mild decrease initially, then returned to their baseline levels. Similarly, a shock to GDP resulted in a substantial increase in energy use, low-carbon energy output, and oil operating surplus, with the effects persisting for several periods, while resource-based revenue and natural gas profit margin showed a moderate decrease initially, followed by a recovery. Also, a shock to low-carbon energy output, natural gas profit margin, and oil operating surplus led to a significant increase in energy use and GDP, with the effects diminishing over time, and a shock to resource-based revenue results in a moderate decrease in energy use, GDP, and low-carbon output, with the effects fading over time.

The results suggest that shocks to energy use, GDP, and oil operating surplus had significant and persistent effects on the variables, indicating strong interdependencies. In contrast, shocks to low-carbon energy out, resource-based revenue, and natural gas profit margins had significant but short-lived effects. The responses to shocks are generally consistent with economic theory, with positive shocks to GDP and oil operating surplus leading to increases in energy use and low-carbon energy output ( Effiong & Hosu, 2025).

**Figure 6.**
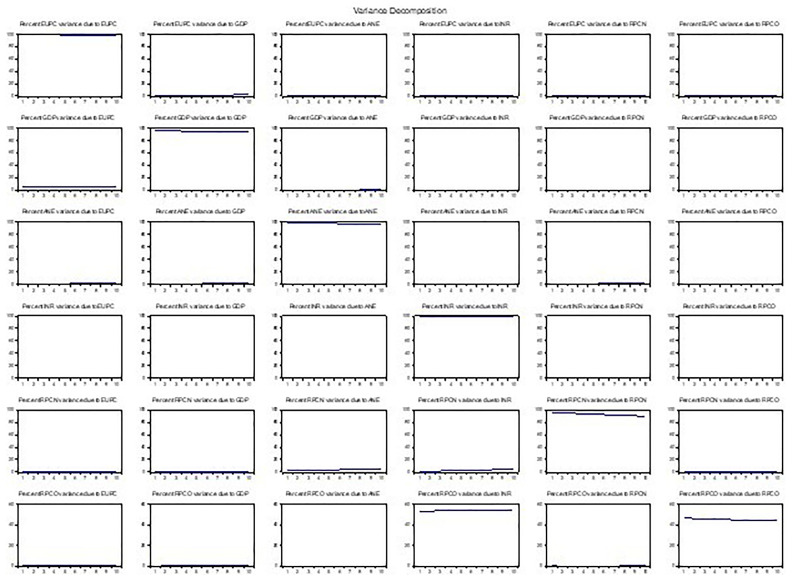
Variance decomposition.

In a VAR model, the Variance Decomposition Analysis (VDA) is usually used to decompose the variance of a variable into the proportions attributed to its shocks and shocks from other variables. As presented in Figure 6, 60% of the variance in energy use is attributed to its shocks, while 20% is attributed to GDP shocks, 10% to low-carbon energy shocks, and 10% to oil operating shocks. The results suggest that energy use and GDP are the most influential variables in the system, with a significant proportion of variance in other variables attributed to their shocks compared to low-carbon energy output and oil operating surplus having significant effects on other variables. In contrast, resource-based revenue and natural gas profit margins had relatively more variance attributed to their shocks. Overall, the findings indicate that shocks to energy use, GDP, and oil operating surplus significantly affect the variables, consistent with the Impulse Response Analysis results.

## Conclusion and recommendations

This study comprehensively analyzes the relationships between energy use, GDP, low-carbon energy output, natural resources, and Africa’s economic performance. The results show that GDP, low-carbon energy output, natural gas profit margin, and oil operating surplus significantly affect energy use, with resource-based revenue having a relatively minor impact. The study also reveals complex dynamics between the variables, with both short-term and long-term effects. Based on the findings, the study reveals strong relationships between energy use and these variables, indicating that increases in GDP, low-carbon energy output, natural gas profit margin, and oil operating surplus are associated with substantial increases in energy consumption. Complex and long-term relationships among the variables exist, consistent with the cointegration analysis. The study also revealed that shocks to energy use, GDP, and oil operating surplus have significant and persistent effects on the variables, indicating strong interdependencies.

Based on the findings of this study, the following recommendations are made:

1. **Diversify energy sources**: Encourage developing and using low-carbon energy sources to reduce dependence on fossil fuels and mitigate climate change.
2. **Invest in renewable energy**: Invest in renewable energy technologies, such as solar and wind power, to increase energy security and reduce greenhouse gas emissions.
3. **Improve energy efficiency**: Implement energy-efficient practices and technologies to reduce energy consumption and promote sustainable development.
4. **Enhance economic development**: Foster economic growth and development by investing in human capital, infrastructure, and innovation.
5. **Promote sustainable resource management**: Implement sustainable resource management practices to ensure the long-term viability of natural resources and minimize environmental impacts.
6. **Develop policies and regulations**: Develop and implement policies and regulations that support the transition to a low-carbon economy and promote sustainable energy use.
7. **Monitor and evaluate progress**: Regularly monitor and evaluate progress towards energy and economic development goals and adjust policies and strategies as needed.

By implementing these recommendations, African countries can promote sustainable energy use, reduce greenhouse gas emissions, and achieve economic development while minimizing environmental impacts. Overall, this study provides a comprehensive analysis of the relationships between energy use, GDP, low-carbon energy output, natural resources, and economic performance in Africa and offers valuable insights for policymakers and stakeholders seeking to promote sustainable energy use and economic development in the region.

## Declarations

### Ethics and consent

Ethical approval and consent were not required.

## Data Availability

Data have been uploaded to the repository (Figshare) via: https://figshare.com/account/home#/data, with a DOI: 10.6084/m9.figshare.28828415, and 10.6084/m9.figshare.28828403 respectively for both figures and datasets. The dataset is in an Excel sheet comprising the above-stated variables for the 46 Sub-Saharan countries used for the study. The dataset has a CC0 Public Domain Dedication license applied The Data used for the study were accessed from: https://www.theglobaleconomy.com. On the webpage, click on download data, select the 46 Sub-Saharan countries. For the variables, under the energy and environment section of the variables selection section, energy use, alternative nuclear energy (low-carbon energy output), GDP, income from natural source (resource based revenue), revenue minus production cost of oil (oil operating surplus), and revenue minus product cost of natural gas (natural gas profit margin) constituted the dependent and explanatory variables used for the analysis in the study.

## Software availability statement

The data analysis for this study was conducted using EViews statistical software, which was instrumental in performing the dynamic panel data estimation, including the Generalized Method of Moments (GMM), Vector Error Correction Model (VECM), and other econometric techniques.

## Software name

EViews 12. EViews is commercially available, and licensed versions can be obtained directly from the official website: https://www.eviews.com.
