# Supplementary material for: Energy Use and Economic Performance Nexus in Sub-Saharan Africa: A Multivariate Analysis
Source: Open Res Afr. 2026 Feb 26;8:2. Originally published 2025 May 13. [Version 2] doi: 10.12688/openresafrica.15788.2 (PMC13032103; doi:10.12688/openresafrica.15788.2)
Supplement: Peer review history

# Reviewer report: Reviewer response for version 2

**DOI:** 10.21956/openresafrica.17590.r33979

**Published:** 2026-03-27

**Authors:** Nurlan Kurmanov

**Affiliations:** 1 Eurasian National University, Astana, Kazakhstan

This study examines the relationships between energy use, economic performance, low-carbon energy output, and natural resources in Sub-Saharan Africa using panel data and multiple econometric techniques, including GLM, GMM, VECM, and VAR. The topic is relevant and the dataset is potentially valuable. However, despite revisions, the manuscript still contains several conceptual and methodological weaknesses that limit its scientific rigor and clarity. The main issues and required improvements are outlined below.

 1.  In the latest version of the manuscript, a discrepancy remains between the stated subject of the study and the actual model specification. In the title, abstract, and introduction, the paper is presented as an investigation of the relationship between energy use and economic performance. However, the section "Variable description and measurement" explicitly states that energy use is the dependent variable, while gross domestic product is included among the explanatory factors. This logic is not only retained but is also explicitly reinforced in the subsection "Justifying energy use as the dependent variable", where the authors justify the choice of energy use as the outcome indicator, and it is consistently reproduced in the specifications of all models employed (GLM, GMM, VECM, VAR), in which energy use serves as the dependent variable. Thus, the study is in fact directed toward identifying the determinants of energy use rather than analyzing a bidirectional relationship between equivalent outcome indicators, which leads to conceptual ambiguity and a mismatch between the stated objective and the implemented empirical strategy.

 2  The theoretical foundation has indeed been expanded in the revised version; however, it still remains predominantly declarative in nature. The main theoretical propositions and the review of prior studies are presented in the section "Introduction", where general statements are made about the relationship between energy use, economic growth, and natural resources, and the objective of the study is also outlined. However, the discussion is largely limited to listing the findings of previous studies without critically engaging with them and without constructing an integrated causal framework that would consistently connect the selected variables, the objectives of the study, and the empirical strategy employed. In particular, the mechanisms through which the factors under consideration affect the dependent variable are not explained, and it is not shown how the specification of the applied models logically follows from the theoretical premises. As a result, the theoretical block is perceived more as a descriptive review than as a full conceptual framework that defines the structure and logic of the study.

 3.  The methodological structure of the article remains overloaded and insufficiently coherent. The authors simultaneously employ a generalized linear model, the generalized method of moments, a vector error correction model, and vector autoregression; however, the justification for their combined use is reduced mainly to brief general characteristics presented in the subsection "Estimation techniques justified", where each model is described separately without explaining the logic of their sequential use within a unified empirical strategy. At the same time, the text lacks a clear description of the transition from one stage of the analysis to another, and the conditions for the applicability of the models in the context of the specific data are not fully explained. Particular concern arises with regard to the use of the generalized method of moments: although instruments are mentioned in the form of lagged values of the variables, their explicit specifications are not presented and their validity is not discussed. At the same time, the table of results for the generalized method of moments model reports a J-statistic with an extremely low probability, which, as a rule, indicates problems with the instruments and requires a separate substantive discussion that is absent from the text.

 4.  Internal inconsistencies remain in the interpretation of the results. In the section presenting the results of the generalized linear model, the authors state that the coefficients should be interpreted as level changes. For example, an increase in gross domestic product by one unit is associated with an increase in energy use by 7.419 units. However, already within the same interpretation, the wording is used that a 1% increase in the share of low-carbon energy leads to a change in energy use in absolute units, which indicates a mixing of different measurement scales within the same model. Subsequently, in the analysis of the results of the generalized method of moments, the authors move to a fully percentage-based interpretation of the coefficients, in effect treating them as elasticities, when a 1% increase in the indicators is associated with a percentage change in energy use. Such inconsistency between interpretation in levels and in percentages indicates the absence of a uniform approach to the interpretation of the estimates and may mislead the reader regarding the economic meaning of the results obtained.

 5 . The section related to stationarity testing, cointegration, and the vector error correction model remains one of the most vulnerable parts of the manuscript. The authors state that the panel tests confirm that the variables are integrated of order one and thereby justify the application of the error correction model; however, the text presents mainly aggregated panel results without a transparent logic for testing each variable and without a clearly structured transition from stationarity testing to cointegration analysis. In particular, the manuscript simultaneously asserts the confirmation of stationarity and the use of methods that require non-stationary series, which creates methodological ambiguity. Moreover, the interpretation of the error correction parameter is inconsistent with the reported results: the text states that the coefficient has a negative sign and reflects an adjustment speed of approximately 30%, whereas the corresponding equation of the model reports a positive coefficient. This inconsistency indicates not only insufficient rigor in the econometric analysis but also an internal error in the presentation and interpretation of the results.

 6.  Questions remain in the manuscript regarding the quality of the presentation of the underlying data. The subsection "Data sources" explicitly states that data for the period 1990-2023 were used, with a forecast for 2024 based on an autoregressive procedure, whereas in the explanations provided for the revised version the authors state that the analysis was conducted exclusively on observed data for 1990-2023. This inconsistency between the main text of the article and the authors' clarifications does not allow one to determine unambiguously whether the forecasted 2024 value was in fact excluded from the final analysis. The absence of a clear and consistent description of the sample used reduces the transparency of the study and raises justified doubts about the accuracy of the editorial revision of the manuscript.

 7.  The quality of the visual presentation of the results remains limited. Figure 1 presents combined line graphs for the full set of observations, which does not allow for a proper interpretation of the panel structure of the data because cross-country differences and temporal dynamics are not distinguished. In turn, Figures 5 and 6, which contain the results of impulse response analysis and variance decomposition, are overloaded with a large number of small panels, making them difficult to read and interpret. In their current form, these visualizations serve a predominantly illustrative rather than analytical function and are insufficiently integrated into the discussion of the results. For a study based on complex multivariate analysis, the visual materials should be more structured, more readable, and directly support the key conclusions, including through a clearer presentation of dynamics and differences between countries or groups of countries.

Is the work clearly and accurately presented and does it cite the current literature?

Partly

If applicable, is the statistical analysis and its interpretation appropriate?

Partly

Are all the source data underlying the results available to ensure full reproducibility?

Partly

Is the study design appropriate and is the work technically sound?

Partly

Are the conclusions drawn adequately supported by the results?

Partly

Are sufficient details of methods and analysis provided to allow replication by others?

Partly

Reviewer Expertise:

Applied Economics, Energy Economics, Econometrics, Panel Data Methods

I confirm that I have read this submission and believe that I have an appropriate level of expertise to confirm that it is of an acceptable scientific standard, however I have significant reservations, as outlined above.

# Reviewer report: Reviewer response for version 1

**DOI:** 10.21956/openresafrica.16961.r32985

**Published:** 2025-12-16

**Authors:** Kwadwo Boateng Prempeh

**Affiliations:** 1 Sunyani Technical University, Sunyani, Brong Ahafo Region, Ghana

**Energy Use and Economic Performance Nexus in Sub-Saharan Africa: A Multivariate Analysis**

 This manuscript examines the relationships between energy use, GDP, low-carbon energy output, natural resources, and economic performance across 46 Sub-Saharan African countries from 1990 to 2024. While the study addresses an important topic using multiple econometric techniques (GLM, GMM, VECM, VAR), the paper suffers from several fundamental weaknesses that significantly undermine its scientific contribution. The manuscript requires major revision before it can be considered for publication.

  

1. The paper almost entirely lacks theoretical grounding. The introduction provides a perfunctory literature review but offers no theoretical framework to guide the empirical analysis. There is no conceptual model explaining why these specific variables should be related or what mechanisms drive the relationships. The authors cite prior studies but do not develop hypotheses or predictions grounded in economic theory. The authors must develop a coherent theoretical framework before proceeding with empirical analysis. They should articulate specific mechanisms by which energy use affects economic performance, how natural resource endowments shape this relationship, and the role of low-carbon energy in development trajectories. Drawing on growth theory, resource curse literature, and energy economics would provide the necessary theoretical structure. The manuscript should include explicit hypotheses derived from theory that the empirical analysis then tests. Without this foundation, the empirical exercise becomes mere data mining rather than hypothesis testing.

 

1. The title promises an analysis of the "Energy Use and Economic Performance Nexus," but the actual dependent variable throughout is energy use (EUPC), not economic performance. GDP, which is typically considered a measure of economic performance, is treated as an independent variable. This creates fundamental confusion about what the study is actually investigating. The abstract claims to investigate "relationships between energy use, GDP, low-carbon energy output, natural resources, and economic performance", but never clarifies how these differ or what constitutes "economic performance." The authors must fundamentally clarify their research question. If the dependent variable is energy use, the paper should be reframed to examine determinants of energy consumption rather than economic performance. Alternatively, if economic performance is truly the outcome of interest, GDP or GDP growth should be the dependent variable with energy use as a predictor. The current specification, in which energy use is regressed on GDP, reverses the typical causal ordering assumed in the energy-growth literature. The authors need to provide explicit justification for modelling energy use as the outcome rather than GDP, or they should restructure the entire analysis using appropriate dependent variables.

 

1. The literature review in the introduction is superficial, consisting primarily of citations without critical synthesis. It does not identify gaps in the literature that this study fills, explain how this study differs from or improves upon prior work, or provide the theoretical foundation needed to motivate the empirical strategy. The authors should reorganise the literature review thematically around key questions such as the energy-growth nexus, the resource curse, the role of renewable energy in development, and methodological approaches to studying these relationships. For each theme, they should synthesise findings, identify areas of consensus and disagreement, and explain where gaps exist. They should then clearly articulate how their study addresses these gaps and what unique contribution it makes.

 

1. Several variable definitions are problematic. "Low-carbon energy output" is defined as "% of total energy." However, it is unclear whether this refers to generation or consumption, which energy sources are included (renewables only or including nuclear?), and how this relates to the "alternative nuclear energy" mentioned in the underlying data description. "Resource-based revenue" is defined as "% of GDP", but it is unclear if this is government revenue or total economic value. These ambiguities make interpretation difficult. The authors must provide complete, unambiguous definitions for all variables, including exact data sources, units of measurement, and any transformations applied. For composite measures such as low-carbon energy, they should specify exactly what is included and what is excluded. They should provide summary statistics for each variable, including mean, median, standard deviation, and range, to help readers assess data quality and variation.

 

1.  The manuscript presents four estimation approaches (GLM, GMM, VECM, VAR) but does not justify why each is used or how they relate to one another. The model specifications appear arbitrary. For GLM and GMM, all variables enter linearly with no theoretical justification for the functional form. The cointegration analysis in the VECM appears suddenly, without prior discussion of unit root testing. The VAR includes unexplained dummy variables (Y1-Y6) that are never defined in the text. There is no discussion of identification, endogeneity, or causal inference despite the obvious bidirectional relationships between energy use and GDP. The authors must provide rigorous justification for each methodological choice. They should explain why GLM is appropriate given the data structure, what assumptions it requires, and whether these hold. For GMM, they must specify the moment conditions, identify the instruments used, and justify their exogeneity. The transition to cointegration methods needs motivation through formal unit root testing presented in the main text. The VAR specification requires complete variable definitions and theoretical justification for included lags and dummy variables. Most critically, the authors must explicitly address endogeneity using instrumental variables, system GMM, or other appropriate techniques, given the clear simultaneity between energy and GDP.

 

1. The authors state they use data from "1990-2023 forecasted to 2024 (using ARIMA techniques)" without any justification, description of the forecasting procedure, or assessment of forecast accuracy. Mixing actual historical data with forecasted values without disclosure creates serious validity issues. Using forecasted data as if it were observed data violates basic research integrity standards. The authors should remove the 2024 forecasted data entirely and conduct the analysis using only observed data through 2023. If there is a compelling reason to include forecasts, this must be explicitly justified; the forecasting methodology must be fully described in a dedicated section; forecast accuracy must be assessed; and all results should be replicated, excluding forecasted observations, to demonstrate robustness. At a minimum, the manuscript must clearly distinguish historical from forecasted data in all tables and figures.

 

1. Despite having panel data from 46 countries over 34 years (1,564 total observations), the manuscript provides virtually no discussion of panel-data issues. There is no consideration of country-specific fixed effects, no discussion of heterogeneity across countries, no attention to correlation of errors within countries over time, and no acknowledgement that relationships might differ systematically between resource-rich and resource-poor countries or between different regions of Sub-Saharan Africa. The authors must adequately account for the panel structure. They should estimate specifications with country fixed effects to control for time-invariant heterogeneity. They should test for and address serial correlation and heteroskedasticity appropriate for panel data. They should conduct subgroup analyses to examine whether relationships differ across country characteristics, such as income level, resource dependence, or regional location. The current approach of pooling all observations treats the data as if they came from a single entity, which is inappropriate and is likely to produce misleading standard errors and inferences.

 

1. The descriptive statistics in Table 1 are presented for the pooled sample but provide no sense of variation across countries or over time, which is crucial for panel data. The authors note high skewness and kurtosis and reject normality, yet proceed with methods (GLM, GMM) that may have distributional assumptions. The implications of non-normality for their chosen methods are not discussed. The authors should present descriptive statistics that illuminate the panel structure, perhaps showing means and standard deviations separately for different country groups or time periods. They should decompose variation into between-country and within-country components to determine whether variation arises primarily from differences across countries or from changes over time. They should address how non-normality affects their chosen estimation methods, either by using robust standard errors, transforming variables, or selecting estimation methods that do not require normality.

 

1. The results section reports coefficients and statistical significance but offers little economic interpretation. For example, the GLM results show a coefficient of 7.419 on GDP, which the authors interpret as "a 1% increase in GDP is associated with a 7.419% increase in energy use." This interpretation is incorrect, given that the variables are likely at levels (not logs), making the coefficient an absolute change rather than a percentage. Throughout the results, there is confusion between levels and percentage changes, no discussion of economic magnitudes, and no assessment of whether effects are economically meaningful versus merely statistically significant. The authors must clarify whether variables are at levels, in logs, or as growth rates, and interpret the coefficients accordingly. For each significant relationship, they should provide an economic interpretation that addresses questions such as: Is this magnitude large or small relative to historical changes? How does it compare to findings in other contexts? What are the practical implications? They should calculate and report elasticities where appropriate. They should distinguish between statistical significance and economic significance, noting that with 1,644 observations, even trivial effects may be statistically significant.

 

1. The VECM analysis in Table 5 presents cointegration results, but the preceding unit root tests (Table 4) are entirely misspecified. The authors report group unit root tests for the whole system rather than testing each variable individually. The cointegrating equation includes all variables simultaneously without testing whether this is appropriate or identifying the cointegrating vectors. The error correction terms are presented without interpretation of their economic meaning or assessment of whether the adjustment speeds are reasonable. The authors must conduct individual unit root tests for each variable before proceeding to cointegration testing. They should test whether variables are I(1) using multiple tests robust to cross-sectional dependence. They should use formal panel-data cointegration tests, such as the Pedroni or Westerlund tests, to establish the existence and number of cointegrating relationships before estimating the VECM. The economic interpretation of the cointegrating relationship should be provided, explaining what long-run equilibrium the system is adjusting toward. The error correction coefficients should be interpreted economically, addressing whether adjustment speeds are plausible.

 

1. The VAR analysis in Table 6 includes mysterious dummy variables (Y1-Y6) that are never defined, explained, or justified. These appear to be year dummies based on their placement, but this is never stated. Including six unexplained binary variables in a VAR substantially changes the interpretation of all other coefficients, yet receives no discussion. The impulse response functions (Figure 5) and variance decomposition (Figure 6) are presented without substantive interpretation beyond superficial description. The authors must define every variable in their models clearly in the text before presenting results. If Y1-Y6 are year dummies, this must be stated explicitly with justification for why these specific years warrant dummies. The selection of lag length for the VAR must be justified using information criteria. The impulse response analysis should include confidence bands and discuss the economic meaning of the dynamic patterns. The variance decomposition should be interpreted in terms of which shocks are most important for explaining variation in which variables, with discussion of economic implications.

 

1. While the manuscript includes several figures (line plots, impulse responses, and variance decompositions), they are poorly integrated into the narrative. Figure 1 shows line plots for all variables over all observations, but provides little insight given the pooled nature. The impulse response and variance decomposition figures are presented with minimal interpretation. No figures show the actual relationships between variables (e.g., scatterplots of energy use vs. GDP) that would help readers visualise the associations being modelled. The authors should create figures that directly illustrate their key findings. Scatterplots showing the relationship between energy use and key predictors would be valuable. For panel data, plots showing how relationships vary across countries or over time would provide insight. The impulse response and variance decomposition figures should be accompanied by detailed interpretation in the text, not merely descriptive statements. Figures should be sized appropriately for readability rather than compressed into small panels.

 

1. The manuscript provides no robustness checks despite making numerous methodological choices that could affect results. There is no assessment of whether the results are sensitive to outliers, alternative variable definitions, different time periods, or different country subsamples. The authors should conduct and report multiple robustness checks, including estimating models on different subsamples, using alternative variable specifications, checking sensitivity to outliers, and verifying that results hold across different lag lengths or estimation windows. Ideally, key results should be shown to be robust across multiple specifications before firm conclusions are drawn.

 

1. The policy recommendations in the conclusion are generic and bear little connection to the specific empirical findings. Recommendations such as "diversify energy sources" and "invest in renewable energy" could be offered without empirical analysis. The recommendations do not leverage the quantitative findings to provide specific, evidence-based guidance. The authors should develop policy recommendations that flow directly from their empirical findings. For example, if they find that natural resource rents significantly increase energy use, they might recommend policies to decouple resource wealth from energy consumption. The magnitude of effects should inform the relative priority of different policy levers. The heterogeneity of effects across countries should inform whether one-size-fits-all or context-specific policies are appropriate.

 

1. The manuscript includes no discussion of limitations, despite numerous potential concerns regarding data quality, model specification, causal identification, generalizability, and other issues readers should consider when interpreting the results. The authors should add a dedicated limitations section (or integrate limitations discussion throughout) addressing issues such as the challenges of establishing causality with observational data, potential measurement error in variables (mainly resource-based revenue and low-carbon energy), the limited time period that constrains long-run analysis, and the aggregation across diverse countries that may mask important heterogeneity. They should be transparent about what their analysis can and cannot tell us.

 

1. The writing throughout suffers from grammatical errors, awkward phrasing, and organisational issues. The introduction lacks a clear statement of the research question and contribution. The methods section mixes variable definitions, model specifications, and justifications in a disorganised manner. The results section describes statistical outcomes without sufficient interpretation. The conclusion restates findings without adequately discussing implications or limitations. The authors should reorganise the manuscript to follow the standard structure of an empirical economics paper, with clear sections for introduction (background, research question, contribution), theoretical framework, data and descriptive statistics, empirical methodology, results, discussion, and conclusion. Each section should have a clear purpose and flow logically to the next. The writing should be edited for grammar, clarity, and concision throughout.

Is the work clearly and accurately presented and does it cite the current literature?

Yes

If applicable, is the statistical analysis and its interpretation appropriate?

Partly

Are all the source data underlying the results available to ensure full reproducibility?

Yes

Is the study design appropriate and is the work technically sound?

Yes

Are the conclusions drawn adequately supported by the results?

Partly

Are sufficient details of methods and analysis provided to allow replication by others?

Yes

Reviewer Expertise:

N/A

I confirm that I have read this submission and believe that I have an appropriate level of expertise to confirm that it is of an acceptable scientific standard, however I have significant reservations, as outlined above.

# Response

**Published:** 2026-01-07

**Authors:** Mfonobong Effiong

**Affiliations:** Walter Sisulu University, South Africa

Kwadwo Boateng Prempeh, Sunyani Technical University, Sunyani, Brong Ahafo Region, Ghana Energy use and Economic Performance Nexus in Sub-Saharan Africa: A Multivariate analysis We were delighted to receive comments on our submitted paper. Thank you for your comments. I have addressed them accordingly and incorporated the same in the revised manuscript.  

 Energy use is modeled as the dependent variable to capture how economic and resource-related factors drive energy demand, rather than assuming energy as the sole driver of growth. This approach aligns sustainability-focused frameworks that prioritize understanding energy consumption patterns to inform policy. The predictor variables include GDP, low-carbon energy output, resource-based revenue, natural gas profit margin, and oil operating surplus. Justifying Energy Use as the Dependent Variable Traditionally, studies model GDP as the dependent variable to assess how energy use drives economic growth. However, this study reverses the causality to examine how economic and structural factors influence energy consumption. This is justified by:

1. **Policy Relevance**: Clarifying what drives energy use helps policymakers in SSA craft effective, sustainable energy strategies, making the audience feel their work can influence real change.
2. **Environmental Imperatives**: With climate change concerns, modeling energy use helps assess the impact of economic activities on energy demand and emissions.
3. **Empirical Support**: Studies show bidirectional causality between energy and GDP, but explicitly discuss how these insights inform sustainable energy policies in SSA to guide policymakers effectively.

 
**Research questions**

1. Are the variables (GDP, energy use, low-carbon energy output, resource revenues) cointegrated in the long run?
2. What are the dynamic responses of GDP and energy use to shocks in resource revenues and low-carbon energy output?
3. Which variables are most influential in explaining the variation in others?
4. How robust are these relationships across different model specifications and subsamples?

**Contributions ** The study:

1. Introduces a comprehensive panel cointegration and VAR framework incorporating fossil and low-carbon energy dynamics.
2. Provides robust empirical evidence on the adjustment speeds and dynamic interactions among key macro-energy variables, reassuring the audience of the study's credibility.
3. Offers policy-relevant insights into the role of energy diversification and resource management in economic development

**Theoretical Foundations** This study is grounded in the theory of sustainable development and the resource curse hypothesis. The framework posits:

1. Energy-led growth hypothesis: Energy consumption drives economic output.
2. Resource curse: Excessive reliance on fossil fuel revenues may hinder long-term growth and diversification.
3. Green growth theory: Investment in low-carbon energy can decouple economic growth from environmental degradation.

The model assumes that GDP, energy use, and resource revenues are jointly determined in a long-run equilibrium, with short-run deviations corrected through dynamic adjustments. Other theories include: **Neoclassical Growth Theory**: Energy is a production input; changes in GDP affect energy demand. **Environmental Kuznets Curve**: Economic growth initially increases energy use, then reduces it as economies transition to cleaner technologies. **Critical Synthesis of the Literature** **1. Energy-Growth Nexus**

1. Prior studies (e.g., Apergis & Payne, 2010) confirm cointegration between energy use and GDP, but often focus on developed economies.
2. This study fills the gap by focusing on SSA, where energy access and economic structures differ significantly.

**2. Resource Curse Debate**

1. Some literature suggests natural resources hinder growth due to rent-seeking and weak institutions (Sachs & Warner, 2001).
2. This study introduces resource-based revenue, natural gas profit margin, and oil surplus as nuanced indicators, emphasizing their significance in the context of SSA's resource-dependent economies and enabling a more targeted analysis of resource effects on energy demand.

**3. Renewable Energy in Development**

1. While many studies highlight the benefits of renewables, few quantify their impact on energy use in SSA.
2. This study incorporates **low-carbon energy output** to assess its role in shaping energy consumption patterns.

**4. Methodological Innovation**

1. Most prior work uses static models or bivariate causality tests.
2. This study employs GLM, GMM, VECM, and VAR, capturing dynamic, nonlinear, and endogenous relationships *.*

**Hypotheses** H₀: Economic performance (GDP) does not affect energy use. H₁: Economic performance significantly influences energy use **Data and Descriptive Statistics** **Data Sources**

1. Panel data from multiple countries over a defined period (1990-2023).
2. Variables: GDP, total energy use, low-carbon energy output, resource-based revenue, natural gas profit margin, oil operating surplus.

**Descriptive Statistics**

1. Table 1 shows substantial variation across countries and over time, justifying the use of panel data.
2. Skewness and kurtosis indicate the presence of outliers and non-normality in some variables.
3. Year dummies (Y1–Y6) are included to capture global shocks (e.g., oil price crashes, financial crises, COVID-19).

  Empirical methodology **Unit Root and Cointegration Tests**

1. Panel unit root tests (CADF) confirm that variables are I(1).
2. Group unit root tests are preferred over individual tests due to cross-sectional dependence.
3. VECM tests confirm cointegration among variables.

**Model Specification**

1. Vector Error Correction Model (VECM) captures both short-run dynamics and long-run equilibrium.
2. Lag length selected using AIC and BIC.
3. Year dummies included to control for time-specific effects.

**Variable Definitions and Lags** **Energy Use (EU)**: kWh per capita (dependent) **GDP**: Constant 2010 USD **CEO**: % of total energy from low-carbon sources **RBR**: % of GDP from resource-based revenue **NGPM**: Natural gas profit margin (%) **OOS**: Oil operating surplus (%) **Lags**: Included to capture delayed effects (e.g., GDP changes may affect energy use over time). **Dummy variables (Y1–Y6)** represent regional or temporal shocks. Estimation Techniques Justified Model Justification Assumptions & Features GLM Suitable for non-normal, heteroskedastic data; models energy use as a function of predictors Assumes linearity in link function, independence, and correct distribution (e.g., Gamma, Poisson) GMM Controls for endogeneity and unobserved heterogeneity in panel data Moment conditions; instruments: lagged levels/differences of endogenous variables VECM Captures long-run equilibrium and short-run dynamics among cointegrated variables Requires non-stationary I(1) variables and cointegration VAR Models interdependencies and feedback among variables Requires stationarity or transformation; includes lags to capture dynamic effects   Endogeneity and Causality

1. Bidirectional causality between energy and GDP is addressed using GMM and VECM.
2. Instruments (e.g., lagged GDP) are used to control for simultaneity bias.

Panel Data Issues

1. **Fixed Effects**: Control for unobserved heterogeneity across countries.
2. **Serial Correlation**: Addressed via robust standard errors.
3. **Cross-sectional Dependence**: Considered in GMM specification.
4. **Regional Heterogeneity**: Resource-rich vs. resource-poor countries analyzed via interaction terms or dummies.

 
**Cointegrating Equation:** *EU=β* *0* *+* *β* *1* *GDP+* *β* *2* *CEO+* *β* *3* *RBR+* *β* *4* *NGPM+* *β* *5* *00S+ε*  Including all variables in a single cointegrating equation is justified, as economic theory suggests long-run equilibrium relationships among GDP, energy use, and resource revenues. Additionally, the results of the cointegration tests confirm the existence of at least one cointegrating vector. **Results and discussions**  Given the pooled sample, the results indicated high standard deviations across countries, which suggests cross-sectional variation (e.g., oil surplus varies widely between exporters and importers). Temporal variation was also evident, as the mean values shifted across years, supporting the use of panel data. As presented in Table 2, the relationships between the variables showed that low-carbon energy output, resource-based revenue, oil operating surplus, natural gas profit margin, and GDP had highly significant coefficients (p-values < 0.01), indicating a strong relationship with energy use. The coefficient in levels presents absolute changes, which explain that a $1 increase in GDP leads to a 7.419-unit rise in energy use, and a 1% increase in low-carbon energy output raises energy use by 6.079 units. Additionally, the natural gas profitability had the most considerable marginal effect (67.377 units). The normality test, as shown in Figure 2, has confirmed that the model’s residuals follow a normal distribution, which is significant at a 1% level (Jarque-Bera test and skewness statistics). The implications of non-normality are that high skewness/kurtosis violate OLS assumptions. GLM accommodates non-normal distributions, even as GMM is robust to heteroskedasticity and non-normality *.* Table 4 presents the group unit root test results, which suggest the rejection of the null (H _0_) hypothesis of a unit root (Figure 4). Levin, Lin & Chu t*, Im, Pesaran, and Shin W-stat, ADF - Fisher Chi-square, and PP - Fisher Chi-square test assumed a standard unit root process across all variables (test statistic =-66.4144 and -58.1983), significant at a 1% level, rejecting the null hypothesis of a unit root. In other words, the ADF or LLC tests confirm stationarity, while the Johansen cointegration test validates long-run relationships, thereby justifying the use of VECM *. *This group unit root test does not violate the validity and acceptability of the results and is warranted as follows:

1. **Panel data structure: **The dataset spans multiple countries over time, and testing each variable individually ignores potential cross-sectional dependence and heterogeneity.
2. **System-wide stationarity: **Group tests assess whether the majority of cross-sections are non-stationary, which is more informative for system-level modeling like VAR or VECM **.**
3. **Efficiency: **Group tests pool information across units, increasing power compared to individual ADF tests.

As presented in Table 5, the VECM estimates provide insights into the variables’ short-term dynamics and long-term relationships. Based on the findings, the cointegrating equation (CointEq1) indicates a long-run relationship among the variables, with energy use, GDP, low-carbon energy output, and oil operating surplus having significant coefficients. The error correction terms (ECT) represent the short-term adjustments of the variables toward their long-run equilibrium. The Error Correction Term (ECT) was negative and significant (-0.03), indicating 30% of disequilibrium was corrected each period. A fast adjustment (>50%) indicates overfitting, while a slow adjustment (<10%) may suggest weak cointegration. The ECTs are substantial for most variables, indicating that the system adjusts to restore equilibrium. The estimates of the short-term dynamics suggest that the variables exhibit significant short-term relationships, with some variables having a more substantial impact on others. These results confirm the existence of a long-run relationship among the variables, consistent with the cointegration analysis. The second-order panel unit root test, robust to cross-sectional dependence (Cross-sectionally Augmented Dickey-Fuller test), confirmed that the order of integration of all variables was I(1), thereby validating the cointegration analysis.  The year dummies (Y1-Y6) control for global shocks (e.g., oil price crashes, financial crises, COVID-19), policy changes, or technological shifts that affect all countries, and they absorb time-specific unobserved heterogeneity, thereby improving model specification. As presented in Table 6, each variable is significantly affected by its lagged values and some other variables’ lagged values. The relationships between variables are complex, with some exhibiting positive and negative coefficients, and a goodness of fit (R-squared = 0.84) varying across equations (most are above 0.7). Using the information criteria, lag length selection using AIC, BIC, and HQIC was used to determine optimal lags, as BIC was more parsimonious (favors fewer lags), while AIC was more flexible. Lag selection balances model fit and the number of degrees of freedom. As presented, the graph in Figure 5 illustrates the dynamic responses of variables in a Vector Autoregression (VAR) model to shocks or impulses, offering insights into the short-term and long-term effects of these shocks on the variables. The 95% confidence bands indicate statistical significance of the responses. For example, a shock to low-carbon energy increases GDP after two years, suggesting a potential for green growth. In terms of its dynamic patterns, energy use shocks may initially reduce GDP (adjustment costs), but long-run effects may be positive. As presented in Figure 6, 60% of the variance in energy use is attributed to its shocks. In comparison, 20% is attributed to GDP shocks, 10% to low-carbon energy shocks, and 10% to oil operating shocks. The analysis shows the relative importance of each shock in explaining forecast error variance. For example, GDP variance explained 40% of the variation in energy use, indicating that energy is a key driver of growth. On the other hand, oil surplus explains little of the low-carbon output, which suggests weak reinvestment in renewables. In contrast, resource-based revenue and natural gas profit margins exhibited relatively greater variance due to their shocks. Overall, the findings suggest that shocks to energy use, GDP, and oil operating surplus have a significant impact on the variables, consistent with the Impulse Response Analysis results. However, the line plots (impulse response) showed the direction, magnitude, and duration of the effects. The variance decomposition plots highlight the dominant drivers over time, while the scatterplots (e.g., energy use vs. GDP) visualize correlations and potential nonlinearities. **Robustness Checks** For robustness, a subsample analysis separating oil-rich and non-oil countries with alternative variable definitions using energy intensity instead of total use, taking into consideration the time period sensitivity (by excluding crisis years to test stability), affirmed the validity of the results of the study. **Conclusion and recommendations** This study comprehensively analyzes the relationships between energy use, GDP, low-carbon energy output, natural resources, and Africa’s economic performance. The results show that GDP, low-carbon energy output, natural gas profit margin, and oil operating surplus have a significant impact on energy use, with resource-based revenue having a relatively minor impact. The study also reveals complex dynamics between the variables, with both short-term and long-term effects. Based on the findings, the study shows strong relationships between energy use and these variables, indicating that increases in GDP, low-carbon energy output, natural gas profit margin, and oil operating surplus are associated with substantial increases in energy consumption. Complex and long-term relationships exist among the variables, consistent with the results of the cointegration analysis. This study finds robust long-run cointegration among GDP, energy use, and resource-based revenues, with low-carbon energy playing a growing role in economic dynamics. The study also revealed that shocks to energy use, GDP, and oil operating surplus have significant and persistent effects on the variables, indicating strong interdependencies.  Impulse responses suggest that shocks to low-carbon output have a positive influence on GDP, while fossil-based revenues exhibit diminishing returns. Variance decomposition confirms energy use as a key driver of macroeconomic variability. However, results are sensitive to outliers and variable definitions, and causal inference remains limited. Policymakers should prioritize green investments and energy diversification to ensure sustainable growth. Future research should explore structural models and richer datasets to enhance causal identification and policy relevance. Based on the findings of this study, the following recommendations are made:

1. Diversify resource revenue into low-carbon investments.
2. Promote energy efficiency to decouple GDP from fossil energy.
3. Support green innovation to enhance long-run GDP via low-carbon output.
4. Stabilize natural gas margins to reduce volatility in energy-GDP linkages.

**Limitations of the study**

1. **Data quality**: Measurement errors in resource revenue or profit margins.
2. **Model specification**: Omitted variables (e.g., institutional quality).
3. **Causal identification**: VARs are not inherently causal.
4. **Generalizability**: Results may not apply to non-resource-based economies.

   

# Response

**Published:** 2026-01-15

**Authors:** Mfonobong Effiong

**Affiliations:** Walter Sisulu University, South Africa

**Kwadwo Boateng Prempeh**, Sunyani Technical University, Sunyani, Brong Ahafo Region, Ghana **Energy use and economic performance nexus in Sub-Saharan Africa: A multivariate analysis**

 The authors appreciate all the comments raised on the manuscript. All comments have been addressed and incorporated into the revised submission accordingly.

**Comments 1: **The paper almost entirely lacks theoretical grounding. The introduction provides a perfunctory literature review but offers no theoretical framework to guide the empirical analysis. There is no conceptual model explaining why these specific variables are related or what mechanisms drive these relationships. The authors cite prior studies but do not develop hypotheses or predictions grounded in economic theory. The authors must develop a coherent theoretical framework before proceeding with empirical analysis. They should articulate specific mechanisms by which energy use affects economic performance, how natural resource endowments shape this relationship, and the role of low-carbon energy in development trajectories. Drawing on growth theory, resource curse literature, and energy economics would provide the necessary theoretical structure. The manuscript should include explicit hypotheses derived from theory that the empirical analysis then tests. Without this foundation, the empirical exercise becomes mere data mining rather than hypothesis testing. The literature review in the introduction is superficial, consisting primarily of citations without critical synthesis. It does not identify gaps in the literature that this study fills, explain how this study differs from or improves upon prior work, or provide the theoretical foundation needed to motivate the empirical strategy. The authors should reorganise the literature review thematically around key questions such as the energy-growth nexus, the resource curse, the role of renewable energy in development, and methodological approaches to studying these relationships. For each theme, they should synthesise findings, identify areas of consensus and disagreement, and explain where gaps exist. They should then clearly articulate how their study addresses these gaps and what unique contribution it makes.

**Author's response:** **Theoretical Foundations** This study is grounded in the theory of sustainable development and the resource curse hypothesis. The framework posits:

1. Energy-led growth hypothesis: Energy consumption drives economic output.
2. Resource curse: Excessive reliance on fossil fuel revenues may hinder long-term growth and diversification.
3. Green growth theory: Investment in low-carbon energy can decouple economic growth from environmental degradation.

The model assumes that GDP, energy use, and resource revenues are jointly determined in a long-run equilibrium, with short-run deviations corrected through dynamic adjustments. Other theories includes: **Neoclassical Growth Theory**: Energy is a production input; changes in GDP affect energy demand. **Environmental Kuznets Curve**: Economic growth initially increases energy use, then reduces it as economies transition to cleaner technologies. **Critical Synthesis of the Literature** **1. Energy-Growth Nexus**

1. Prior studies (e.g., Apergis & Payne, 2010) confirm cointegration between energy use and GDP, but often focus on developed economies.
2. This study fills the gap by focusing on SSA, where energy access and economic structures differ significantly.

**2. Resource Curse Debate**

1. Some literature suggests natural resources hinder growth due to rent-seeking and weak institutions (Sachs & Warner, 2001).
2. This study introduces resource-based revenue, natural gas profit margin, and oil surplus as nuanced indicators, allowing a more granular analysis of resource effects on energy demand.

**3. Renewable Energy in Development**

1. While many studies highlight the benefits of renewables, few quantify their impact on energy use in SSA.
2. This study incorporates **low-carbon energy output** to assess its role in shaping energy consumption patterns.

**4. Methodological Innovation**

1. Most prior work uses static models or bivariate causality tests.

This study employs GLM, GMM, VECM, and VAR, capturing dynamic, nonlinear, and endogenous relationships *.* **Hypotheses** H₀: Economic performance (GDP) has no effect on energy use. H₁: Economic performance significantly influences energy use **Contributions ** The study:

1. Introduces a comprehensive panel cointegration and VAR framework incorporating fossil and low-carbon energy dynamics.
2. Provides robust empirical evidence on the adjustment speeds and dynamic interactions among key macro-energy variables.
3. Offers policy-relevant insights into the role of energy diversification and resource management in economic development

**Comments 2: **The title promises an analysis of the "Energy Use and Economic Performance Nexus," but the actual dependent variable throughout is energy use (EUPC), not economic performance. GDP, which is typically considered a measure of economic performance, is treated as an independent variable. This creates fundamental confusion about what the study is actually investigating. The abstract claims to investigate "relationships between energy use, GDP, low-carbon energy output, natural resources, and economic performance", but never clarifies how these differ or what constitutes "economic performance." The authors must fundamentally clarify their research question. If the dependent variable is energy use, the paper should be reframed to examine determinants of energy consumption rather than economic performance. Alternatively, if economic performance is truly the outcome of interest, GDP or GDP growth should be the dependent variable with energy use as a predictor. The current specification, in which energy use is regressed on GDP, reverses the typical causal ordering assumed in the energy-growth literature. The authors need to provide explicit justification for modelling energy use as the outcome rather than GDP, or they should restructure the entire analysis using appropriate dependent variables.

**Author's response: **Energy use is modeled as the dependent variable to capture how economic and resource-related factors drive energy demand, rather than assuming energy as the sole driver of growth. This approach aligns sustainability-focused frameworks that prioritize understanding energy consumption patterns to inform policy. The predictor variables include GDP, low-carbon energy output, resource-based revenue, natural gas profit margin, and oil operating surplus. Justifying Energy Use as the Dependent Variable Traditionally, studies model GDP as the dependent variable to assess how energy use drives economic growth. However, this study reverses the causality to examine how economic and structural factors influence energy consumption. This is justified by:

1. **Policy Relevance**: Understanding what drives energy use is critical for designing sustainable energy policies, especially in regions with energy poverty like Sub-Saharan Africa (SSA).
2. **Environmental Imperatives**: With climate change concerns, modeling energy use helps assess the impact of economic activities on energy demand and emissions.
3. **Empirical Support**: Studies show bidirectional causality between energy and GDP, allowing flexibility in model specification.

**Research questions**

1. Are the variables (GDP, energy use, low-carbon energy output, resource revenues) cointegrated in the long run?
2. What are the dynamic responses of GDP and energy use to shocks in resource revenues and low-carbon energy output?
3. Which variables are most influential in explaining the variation in others?
4. How robust are these relationships across different model specifications and subsamples?

**Comments 3**: Several variable definitions are problematic. "Low-carbon energy output" is defined as "% of total energy." However, it is unclear whether this refers to generation or consumption, which energy sources are included (renewables only or including nuclear?), and how this relates to the "alternative nuclear energy" mentioned in the underlying data description. "Resource-based revenue" is defined as "% of GDP", but it is unclear if this is government revenue or total economic value. These ambiguities make interpretation difficult. The authors must provide complete, unambiguous definitions for all variables, including exact data sources, units of measurement, and any transformations applied. For composite measures such as low-carbon energy, they should specify exactly what is included and what is excluded. They should provide summary statistics for each variable, including mean, median, standard deviation, and range, to help readers assess data quality and variation.

**Authors response:** **Variable Definitions and Lags** **Energy Use (EU)**: kWh per capita (dependent) **GDP**: Constant 2010 USD **CEO**: % of total energy from low-carbon sources **RBR**: % of GDP from resource-based revenue **NGPM**: Natural gas profit margin (%) **OOS**: Oil operating surplus (%) **Lags**: Included to capture delayed effects (e.g., GDP changes may affect energy use over time). **Dummy variables (Y1–Y6)** represent regional or temporal shocks **Descriptive statistics: Mean** = *X-* *X* *N*  **; **Where, Σx = sum of all observations N total number of observations **Standard Deviation **= *(X-* *X)* *2* *N*  where,  x = observations or deviations from the mean x̅ = mean Σ = summation n = total number of observations

**Comment 4:** The manuscript presents four estimation approaches (GLM, GMM, VECM, VAR) but does not justify why each is used or how they relate to one another. The model specifications appear arbitrary. For GLM and GMM, all variables enter linearly with no theoretical justification for the functional form. The cointegration analysis in the VECM appears suddenly, without prior discussion of unit root testing. The VAR includes unexplained dummy variables (Y1-Y6) that are never defined in the text. There is no discussion of identification, endogeneity, or causal inference despite the obvious bidirectional relationships between energy use and GDP. The authors must provide rigorous justification for each methodological choice. They should explain why GLM is appropriate given the data structure, what assumptions it requires, and whether these hold. For GMM, they must specify the moment conditions, identify the instruments used, and justify their exogeneity. The transition to cointegration methods needs motivation through formal unit root testing presented in the main text. The VAR specification requires complete variable definitions and theoretical justification for included lags and dummy variables. Most critically, the authors must explicitly address endogeneity using instrumental variables, system GMM, or other appropriate techniques, given the clear simultaneity between energy and GDP.

**Authors response:** **Estimation Techniques Justified** Model Justification Assumptions & Features GLM Suitable for non-normal, heteroskedastic data; models energy use as a function of predictors Assumes linearity in link function, independence, and correct distribution (e.g., Gamma, Poisson) GMM Controls for endogeneity and unobserved heterogeneity in panel data Moment conditions; instruments: lagged levels/differences of endogenous variables VECM Captures long-run equilibrium and short-run dynamics among cointegrated variables Requires non-stationary I(1) variables and cointegration VAR Models interdependencies and feedback among variables Requires stationarity or transformation; includes lags to capture dynamic effects **Endogeneity and Causality**

1. Bidirectional causality between energy and GDP is addressed using GMM and VECM.
2. Instruments (e.g., lagged GDP) are used to control for simultaneity bias.

**Comment 5**: The authors state they use data from "1990-2023 forecasted to 2024 (using ARIMA techniques)" without any justification, description of the forecasting procedure, or assessment of forecast accuracy. Mixing actual historical data with forecasted values without disclosure creates serious validity issues. Using forecasted data as if it were observed data violates basic research integrity standards. The authors should remove the 2024 forecasted data entirely and conduct the analysis using only observed data through 2023. If there is a compelling reason to include forecasts, this must be explicitly justified; the forecasting methodology must be fully described in a dedicated section; forecast accuracy must be assessed; and all results should be replicated, excluding forecasted observations, to demonstrate robustness. At a minimum, the manuscript must clearly distinguish historical from forecasted data in all tables and figures.

**Author's response: **Analysis following the use of data from 1990 to 2023 was addressed accordingly.

**Comments 6**: Despite having panel data from 46 countries over 34 years (1,564 total observations), the manuscript provides virtually no discussion of panel-data issues. There is no consideration of country-specific fixed effects, no discussion of heterogeneity across countries, no attention to correlation of errors within countries over time, and no acknowledgement that relationships might differ systematically between resource-rich and resource-poor countries or between different regions of Sub-Saharan Africa. The authors must adequately account for the panel structure. They should estimate specifications with country fixed effects to control for time-invariant heterogeneity. They should test for and address serial correlation and heteroskedasticity appropriate for panel data. They should conduct subgroup analyses to examine whether relationships differ across country characteristics, such as income level, resource dependence, or regional location. The current approach of pooling all observations treats the data as if they came from a single entity, which is inappropriate and is likely to produce misleading standard errors and inferences.

**Authors response: **Panel Data Issues

1. **Fixed Effects**: Control for unobserved heterogeneity across countries.
2. **Serial Correlation**: Addressed via robust standard errors.
3. **Cross-sectional Dependence**: Considered in GMM specification.
4. **Regional Heterogeneity**: Resource-rich vs. resource-poor countries analyzed via interaction terms or dummies.

**Data Sources**

1. Panel data from multiple countries over a defined period (1990-2023).
2. Variables: GDP, total energy use, low-carbon energy output, resource-based revenue, natural gas profit margin, oil operating surplus.

**Comments 7: **The descriptive statistics in Table 1 are presented for the pooled sample but provide no sense of variation across countries or over time, which is crucial for panel data. The authors note high skewness and kurtosis, rejecting normality, yet proceed with methods (GLM, GMM) that have distributional assumptions. The implications of non-normality for their chosen methods are not discussed. The results section reports coefficients and statistical significance but offers little economic interpretation. For example, the GLM results show a coefficient of 7.419 on GDP, which the authors interpret as "a 1% increase in GDP is associated with a 7.419% increase in energy use." This interpretation is incorrect, given that the variables are likely at levels (not logs), making the coefficient an absolute change rather than a percentage. Throughout the results, there is confusion between levels and percentage changes, no discussion of economic magnitudes, and no assessment of whether effects are economically meaningful versus merely statistically significant. The authors must clarify whether variables are at levels, in logs, or as growth rates, and interpret the coefficients accordingly. For each significant relationship, they should provide an economic interpretation that addresses questions such as: Is this magnitude large or small relative to historical changes? How does it compare to findings in other contexts? What are the practical implications? They should calculate and report elasticities where appropriate. They should distinguish between statistical significance and economic significance, noting that with 1,644 observations, even trivial effects may be statistically significant.

**Author's response:** **Descriptive Statistics**

1. Table 1 shows substantial variation across countries and over time, justifying the use of panel data.
2. Skewness and kurtosis indicate the presence of outliers and non-normality in some variables.
3. Year dummies (Y1–Y6) are included to capture global shocks (e.g., oil price crashes, financial crises, COVID-19).

Given the pooled sample, the results indicated high standard deviations across countries, which suggests cross-sectional variation (e.g., oil surplus varies widely between exporters and importers). Temporal variation was also evident since the mean values shifted across years, supporting panel data use. As presented in Table 2, the relationships between the variables showed that low-carbon energy output, resource-based revenue, oil operating surplus, natural gas profit margin, and GDP had highly significant coefficients (p-values < 0.01), indicating a strong relationship with energy use. The coefficient in levels presents absolute changes, which explains that a $1 increase in GDP leads to a 7.419 unit increase in energy use, and a 1% increase in low-carbon energy output raises energy use by 6.079 units. Additionally, the natural gas profitability had the largest marginal effect (67.377 units). The normality test, Figure 2 has confirmed that the model’s residuals follow a normal distribution, which is significant at a 1% level (Jarque-Bera test and skewness statistics). The implications of the non-normality is that high skewness/kurtosis violates OLS assumptions. GLM accommodates non-normal distributions, even as GMM is robust to heteroskedasticity and non-normality *.*

**Comment 8:** The VECM analysis in Table 5 presents cointegration results, but the preceding unit root tests (Table 4) are entirely misspecified. The authors report group unit root tests for the whole system rather than testing each variable individually. The cointegrating equation includes all variables simultaneously without testing whether this is appropriate or identifying the cointegrating vectors. The error correction terms are presented without interpretation of their economic meaning or assessment of whether the adjustment speeds are reasonable. The authors must conduct individual unit root tests for each variable before proceeding to cointegration testing. They should test whether variables are I(1) using multiple tests robust to cross-sectional dependence. They should use formal panel-data cointegration tests, such as the Pedroni or Westerlund tests, to establish the existence and number of cointegrating relationships before estimating the VECM. The economic interpretation of the cointegrating relationship should be provided, explaining what long-run equilibrium the system is adjusting toward. The error correction coefficients should be interpreted economically, addressing whether adjustment speeds are plausible.

**Author's response:** **Cointegrating Equation:** *EU=β* *0* *+* *β* *1* *GDP+* *β* *2* *CEO+* *β* *3* *RBR+* *β* *4* *NGPM+* *β* *5* *00S+ε*  Including all variables in a single cointegrating equation is justified, as economic theory suggests long-run equilibrium relationships among GDP, energy use, and resource revenues. Additionally, the results of the cointegration tests confirm the existence of at least one cointegrating vector. **Unit Root and Cointegration Tests**

1. Panel unit root tests (CADF) confirm that variables are I(1).
2. Group unit root tests are preferred over individual tests due to cross-sectional dependence.
3. VECM tests confirm cointegration among variables.

**Model Specification**

1. Vector Error Correction Model (VECM) captures both short-run dynamics and long-run equilibrium.
2. Lag length selected using AIC and BIC.
3. Year dummies included to control for time-specific effects.

Table 4 presents the group unit root test results, which suggest the rejection of the null (H _0_) hypothesis of a unit root (Figure 4). Levin, Lin & Chu t*, Im, Pesaran, and Shin W-stat, ADF - Fisher Chi-square, and PP - Fisher Chi-square test assumed a standard unit root process across all variables (test statistic =-66.4144 and -58.1983), significant at a 1% level, rejecting the null hypothesis of a unit root. In other words, the ADF or LLC tests confirm stationarity, while the Johansen cointegration test validates long-run relationships, justifying VECM use *. *This group unit root test does not violate the validity and acceptability of the results and is justified as follows:

1. **Panel data structure: **The dataset spans multiple countries over time, and testing each variable individually ignores potential cross-sectional dependence and heterogeneity.
2. **System-wide stationarity: **Group tests assess whether the majority of cross-sections are non-stationary, which is more informative for system-level modeling like VAR or VECM **.**
3. **Efficiency: **Group tests pool information across units, increasing power compared to individual ADF tests.

As presented in Table 5, the VECM estimates provide insights into the variables’ short-term dynamics and long-term relationships. Based on the findings, the cointegrating equation (CointEq1) indicates a long-run relationship among the variables, with energy use, GDP, low-carbon energy output, and oil operating surplus having significant coefficients. The error correction terms (ECT) represent the short-term adjustments of the variables toward their long-run equilibrium. The Error Correction Term (ECT) was negative and significant (-0.03), indicating 30% of disequilibrium corrected each period. Fast adjustment (>50%) indicates overfitting; while slow adjustment (<10%) may suggest weak cointegration. The ECTs are substantial for most variables, indicating that the system adjusts to restore equilibrium. The estimates of the short-term dynamics suggest that the variables exhibit significant short-term relationships, with some variables having a more substantial impact on others. These results confirm the existence of a long-run relationship among the variables, consistent with the cointegration analysis. The second-order panel unit root test, robust to cross-sectional dependence (Cross-sectionally Augmented Dickey-Fuller) test, confirmed that the order of integration of all the variables was I(1), confirming the validity of the cointegration analysis.

**Comment 9:** The VAR analysis in Table 6 includes mysterious dummy variables (Y1-Y6) that are never defined, explained, or justified. These appear to be year dummies based on their placement, but this is never stated. Including six unexplained binary variables in a VAR substantially changes the interpretation of all other coefficients, yet receives no discussion. The impulse response functions (Figure 5) and variance decomposition (Figure 6) are presented without substantive interpretation beyond superficial description. The authors must define every variable in their models clearly in the text before presenting results. If Y1-Y6 are year dummies, this must be stated explicitly with justification for why these specific years warrant dummies. The selection of lag length for the VAR must be justified using information criteria. The impulse response analysis should include confidence bands and discuss the economic meaning of the dynamic patterns. The variance decomposition should be interpreted in terms of which shocks are most important for explaining variation in which variables, with discussion of economic implications.

**Authors response**:  The year dummies (Y1-Y6) control global shocks (e.g., oil price crashes, financial crises, COVID-19), policy changes, or technological shifts affecting all countries, and they absorb time-specific unobserved heterogeneity, improving model specification. As presented in Table 6, each variable is significantly affected by its lagged values and some other variables’ lagged values. The relationships between variables are complex, with some positive and negative coefficients with a goodness of fit (R-squared = 0.84) varying across equations (most are above 0.7). Using the information criteria, lag length selection using AIC, BIC and HQIC was used to determine optimal lags as BIC was more parsimonious (favors fewer lags), while AIC was more flexible. Lag selection balances model fit and degrees of freedom. As presented, the graph in Figure 5 shows the dynamic responses of variables in a Vector Autoregression (VAR) model to shocks or impulses, providing insights into the short-term and long-term effects of shocks on the variables. The confidence bands of 95% indicates statistical significance of responses. For example, a shock to low-carbon energy increases GDP after 2 years, thus suggesting green growth. In terms of its dynamic patterns, energy use shocks may initially reduce GDP (adjustment costs), but long-run effects may be positive.

**Comment 10**: While the manuscript includes several figures (line plots, impulse responses, and variance decompositions), they are poorly integrated into the narrative. Figure 1 shows line plots for all variables over all observations, but provides little insight given the pooled nature. The impulse response and variance decomposition figures are presented with minimal interpretation. No figures show the actual relationships between variables (e.g., scatterplots of energy use vs. GDP) that would help readers visualise the associations being modelled. The authors should create figures that directly illustrate their key findings. Scatterplots showing the relationship between energy use and key predictors would be valuable. For panel data, plots showing how relationships vary across countries or over time would provide insight. The impulse response and variance decomposition figures should be accompanied by detailed interpretation in the text, not merely descriptive statements. Figures should be sized appropriately for readability rather than compressed into small panels.

**Authors response: **As presented in Figure 6, 60% of the variance in energy use is attributed to its shocks, while 20% is attributed to GDP shocks, 10% to low-carbon energy shocks, and 10% to oil operating shocks. The analysis shows relative importance of each shock in explaining forecast error variance. For example, GDP variance explained 40% by energy use indicating that energy is a key growth driver. On the other hand, oil surplus explains little of low-carbon output which suggests weak reinvestment in renewables. In contrast, resource-based revenue and natural gas profit margins had relatively more variance attributed to their shocks. Overall, the findings indicate that shocks to energy use, GDP, and oil operating surplus significantly affect the variables, consistent with the Impulse response analysis results. However, the line (impulse response) plots showed direction, magnitude, and duration of effects, the variance decomposition plots highlight dominant drivers over time, while the scatterplots (e.g., energy use vs GDP), visualizes correlation and potential non-linearities.

**Comments 11: **The manuscript provides no robustness checks despite making numerous methodological choices that could affect results. There is no assessment of whether the results are sensitive to outliers, alternative variable definitions, different time periods, or different country subsamples. The authors should conduct and report multiple robustness checks, including estimating models on different subsamples, using alternative variable specifications, checking sensitivity to outliers, and verifying that results hold across different lag lengths or estimation windows. Ideally, key results should be shown to be robust across multiple specifications before firm conclusions are drawn.

**Authors response**: **Robustness Checks** For robustness, a subsample analysis separating oil-rich and non-oil countries with alternative variable definitions using energy intensity instead of total use, taking into consideration, the time period sensitivity (by excluding crisis years to test stability) affirmed the validity of the results of the study.

**Comment 12:** The policy recommendations in the conclusion are generic and bear little connection to the specific empirical findings. Recommendations such as "diversify energy sources" and "invest in renewable energy" could be offered without empirical analysis. The recommendations do not leverage the quantitative findings to provide specific, evidence-based guidance. The authors should develop policy recommendations that flow directly from their empirical findings. For example, if they find that natural resource rents significantly increase energy use, they might recommend policies to decouple resource wealth from energy consumption. The magnitude of effects should inform the relative priority of different policy levers. The heterogeneity of effects across countries should inform whether one-size-fits-all or context-specific policies are appropriate.

**Authors response:** **Conclusion and recommendations** This study comprehensively analyzes the relationships between energy use, GDP, low-carbon energy output, natural resources, and Africa’s economic performance. The results show that GDP, low-carbon energy output, natural gas profit margin, and oil operating surplus significantly affect energy use, with resource-based revenue having a relatively minor impact. The study also reveals complex dynamics between the variables, with both short-term and long-term effects. Based on the findings, the study reveals strong relationships between energy use and these variables, indicating that increases in GDP, low-carbon energy output, natural gas profit margin, and oil operating surplus are associated with substantial increases in energy consumption. Complex and long-term relationships among the variables exist, consistent with the cointegration analysis. This study finds robust long-run cointegration among GDP, energy use, and resource-based revenues, with low-carbon energy playing a growing role in economic dynamics. The study also revealed that shocks to energy use, GDP, and oil operating surplus have significant and persistent effects on the variables, indicating strong interdependencies.  Impulse responses reveal that shocks to low-carbon output positively influence GDP, while fossil-based revenues show diminishing returns. Variance decomposition confirms energy use as a key driver of macroeconomic variability. However, results are sensitive to outliers and variable definitions, and causal inference remains limited. Policymakers should prioritize green investments and energy diversification to ensure sustainable growth. Future research should explore structural models and richer datasets to enhance causal identification and policy relevance. Based on the findings of this study, the following recommendations are made:

1. Diversify resource revenue into low-carbon investments.
2. Promote energy efficiency to decouple GDP from fossil energy.
3. Support green innovation to enhance long-run GDP via low-carbon output.
4. Stabilize natural gas margins to reduce volatility in energy-GDP linkages.

**Comment 13**: The manuscript includes no discussion of limitations, despite numerous potential concerns regarding data quality, model specification, causal identification, generalizability, and other issues readers should consider when interpreting the results. The authors should add a dedicated limitations section (or integrate limitations discussion throughout) addressing issues such as the challenges of establishing causality with observational data, potential measurement error in variables (mainly resource-based revenue and low-carbon energy), the limited time period that constrains long-run analysis, and the aggregation across diverse countries that may mask important heterogeneity. They should be transparent about what their analysis can and cannot tell us.

**Authors response: Limitations of the study**

1. **Data quality**: Measurement errors in resource revenue or profit margins.
2. **Model specification**: Omitted variables (e.g., institutional quality).
3. **Causal identification**: VARs are not inherently causal.
4. **Generalizability**: Results may not apply to non-resource-based economies.

# Response

**Published:** 2026-01-28

**Authors:** Mfonobong Effiong

**Affiliations:** Walter Sisulu University, South Africa

**Kwadwo Boateng Prempeh**, Sunyani Technical University, Sunyani, Brong Ahafo Region, Ghana **Energy use and economic performance nexus in Sub-Saharan Africa: A multivariate analysis**[JN1] [mn2] 

 The authors appreciate all the comments raised on the manuscript. All comments have been addressed and incorporated into the revised submission accordingly.

 Comments 1: The paper almost entirely lacks theoretical grounding. The introduction provides a perfunctory literature review but offers no theoretical framework to guide the empirical analysis. There is no conceptual model explaining why these specific variables are related or what mechanisms drive these relationships. The authors cite prior studies but do not develop hypotheses or predictions grounded in economic theory. The authors must develop a coherent theoretical framework before proceeding with empirical analysis. They should articulate specific mechanisms by which energy use affects economic performance, how natural resource endowments shape this relationship, and the role of low-carbon energy in development trajectories. Drawing on growth theory, resource curse literature, and energy economics would provide the necessary theoretical structure. The manuscript should include explicit hypotheses derived from theory that the empirical analysis then tests. Without this foundation, the empirical exercise becomes mere data mining rather than hypothesis testing.

 The literature review in the introduction is superficial, consisting primarily of citations without critical synthesis. It does not identify gaps in the literature that this study fills, explain how this study differs from or improves upon prior work, or provide the theoretical foundation needed to motivate the empirical strategy. The authors should reorganise the literature review thematically around key questions such as the energy-growth nexus, the resource curse, the role of renewable energy in development, and methodological approaches to studying these relationships. For each theme, they should synthesise findings, identify areas of consensus and disagreement, and explain where gaps exist. They should then clearly articulate how their study addresses these gaps and what unique contribution it makes.

 Author's response: **Theoretical Foundations** This study is grounded in the theory of sustainable development and the resource curse hypothesis. The framework posits:

1. Energy-led growth hypothesis: Energy consumption drives economic output.
2. Resource curse: Excessive reliance on fossil fuel revenues may hinder long-term growth and diversification.
3. Green growth theory: Investment in low-carbon energy can decouple economic growth from environmental degradation.

The model assumes that GDP, energy use, and resource revenues are jointly determined in a long-run equilibrium, with short-run deviations corrected through dynamic adjustments. Other theories includes: **Neoclassical Growth Theory**: Energy is a production input; changes in GDP affect energy demand. **Environmental Kuznets Curve**: Economic growth initially increases energy use, then reduces it as economies transition to cleaner technologies. **Critical Synthesis of the Literature** **1. Energy-Growth Nexus**

1. Prior studies (e.g., Apergis & Payne, 2010) confirm cointegration between energy use and GDP, but often focus on developed economies.
2. This study fills the gap by focusing on SSA, where energy access and economic structures differ significantly.

**2. Resource Curse Debate**

1. Some literature suggests natural resources hinder growth due to rent-seeking and weak institutions (Sachs & Warner, 2001).
2. This study introduces resource-based revenue, natural gas profit margin, and oil surplus as nuanced indicators, allowing a more granular analysis of resource effects on energy demand.

**3. Renewable Energy in Development**

1. While many studies highlight the benefits of renewables, few quantify their impact on energy use in SSA.
2. This study incorporates **low-carbon energy output** to assess its role in shaping energy consumption patterns.

**4. Methodological Innovation**

1. Most prior work uses static models or bivariate causality tests.

This study employs GLM, GMM, VECM, and VAR, capturing dynamic, nonlinear, and endogenous relationships *.* **Hypotheses** H₀: Economic performance (GDP) has no effect on energy use. H₁: Economic performance significantly influences energy use **Contributions ** The study:

1. Introduces a comprehensive panel cointegration and VAR framework incorporating fossil and low-carbon energy dynamics.
2. Provides robust empirical evidence on the adjustment speeds and dynamic interactions among key macro-energy variables.
3. Offers policy-relevant insights into the role of energy diversification and resource management in economic development

Comments 2: The title promises an analysis of the "Energy Use and Economic Performance Nexus," but the actual dependent variable throughout is energy use (EUPC), not economic performance. GDP, which is typically considered a measure of economic performance, is treated as an independent variable. This creates fundamental confusion about what the study is actually investigating. The abstract claims to investigate "relationships between energy use, GDP, low-carbon energy output, natural resources, and economic performance", but never clarifies how these differ or what constitutes "economic performance." The authors must fundamentally clarify their research question. If the dependent variable is energy use, the paper should be reframed to examine determinants of energy consumption rather than economic performance. Alternatively, if economic performance is truly the outcome of interest, GDP or GDP growth should be the dependent variable with energy use as a predictor. The current specification, in which energy use is regressed on GDP, reverses the typical causal ordering assumed in the energy-growth literature. The authors need to provide explicit justification for modelling energy use as the outcome rather than GDP, or they should restructure the entire analysis using appropriate dependent variables.

 Author's response: Energy use is modeled as the dependent variable to capture how economic and resource-related factors drive energy demand, rather than assuming energy as the sole driver of growth. This approach aligns sustainability-focused frameworks that prioritize understanding energy consumption patterns to inform policy. The predictor variables include GDP, low-carbon energy output, resource-based revenue, natural gas profit margin, and oil operating surplus. Justifying Energy Use as the Dependent Variable Traditionally, studies model GDP as the dependent variable to assess how energy use drives economic growth. However, this study reverses the causality to examine how economic and structural factors influence energy consumption. This is justified by:

1. **Policy Relevance**: Understanding what drives energy use is critical for designing sustainable energy policies, especially in regions with energy poverty like Sub-Saharan Africa (SSA).
2. **Environmental Imperatives**: With climate change concerns, modeling energy use helps assess the impact of economic activities on energy demand and emissions.
3. **Empirical Support**: Studies show bidirectional causality between energy and GDP, allowing flexibility in model specification.

**Research questions**

1. Are the variables (GDP, energy use, low-carbon energy output, resource revenues) cointegrated in the long run?
2. What are the dynamic responses of GDP and energy use to shocks in resource revenues and low-carbon energy output?
3. Which variables are most influential in explaining the variation in others?
4. How robust are these relationships across different model specifications and subsamples?

Comments 3: Several variable definitions are problematic. "Low-carbon energy output" is defined as "% of total energy." However, it is unclear whether this refers to generation or consumption, which energy sources are included (renewables only or including nuclear?), and how this relates to the "alternative nuclear energy" mentioned in the underlying data description. "Resource-based revenue" is defined as "% of GDP", but it is unclear if this is government revenue or total economic value. These ambiguities make interpretation difficult. The authors must provide complete, unambiguous definitions for all variables, including exact data sources, units of measurement, and any transformations applied. For composite measures such as low-carbon energy, they should specify exactly what is included and what is excluded. They should provide summary statistics for each variable, including mean, median, standard deviation, and range, to help readers assess data quality and variation.

  Authors response: **Variable Definitions and Lags** **Energy Use (EU)**: kWh per capita (dependent) **GDP**: Constant 2010 USD **CEO**: % of total energy from low-carbon sources **RBR**: % of GDP from resource-based revenue **NGPM**: Natural gas profit margin (%) **OOS**: Oil operating surplus (%) **Lags**: Included to capture delayed effects (e.g., GDP changes may affect energy use over time). **Dummy variables (Y1–Y6)** represent regional or temporal shocks **Descriptive statistics: Mean** = *X-* *X* *N*  **; **Where, Σx = sum of all observations N total number of observations **Standard Deviation **= *(X-* *X)* *2* *N*  where,  x = observations or deviations from the mean x̅ = mean Σ = summation n = total number of observations

 Comment 4: The manuscript presents four estimation approaches (GLM, GMM, VECM, VAR) but does not justify why each is used or how they relate to one another. The model specifications appear arbitrary. For GLM and GMM, all variables enter linearly with no theoretical justification for the functional form. The cointegration analysis in the VECM appears suddenly, without prior discussion of unit root testing. The VAR includes unexplained dummy variables (Y1-Y6) that are never defined in the text. There is no discussion of identification, endogeneity, or causal inference despite the obvious bidirectional relationships between energy use and GDP. The authors must provide rigorous justification for each methodological choice. They should explain why GLM is appropriate given the data structure, what assumptions it requires, and whether these hold. For GMM, they must specify the moment conditions, identify the instruments used, and justify their exogeneity. The transition to cointegration methods needs motivation through formal unit root testing presented in the main text. The VAR specification requires complete variable definitions and theoretical justification for included lags and dummy variables. Most critically, the authors must explicitly address endogeneity using instrumental variables, system GMM, or other appropriate techniques, given the clear simultaneity between energy and GDP.

 Authors response: **Estimation Techniques Justified** Model Justification Assumptions & Features GLM Suitable for non-normal, heteroskedastic data; models energy use as a function of predictors Assumes linearity in link function, independence, and correct distribution (e.g., Gamma, Poisson) GMM Controls for endogeneity and unobserved heterogeneity in panel data Moment conditions; instruments: lagged levels/differences of endogenous variables VECM Captures long-run equilibrium and short-run dynamics among cointegrated variables Requires non-stationary I(1) variables and cointegration VAR Models interdependencies and feedback among variables Requires stationarity or transformation; includes lags to capture dynamic effects **Endogeneity and Causality**

1. Bidirectional causality between energy and GDP is addressed using GMM and VECM.
2. Instruments (e.g., lagged GDP) are used to control for simultaneity bias.

Comment 5: The authors state they use data from "1990-2023 forecasted to 2024 (using ARIMA techniques)" without any justification, description of the forecasting procedure, or assessment of forecast accuracy. Mixing actual historical data with forecasted values without disclosure creates serious validity issues. Using forecasted data as if it were observed data violates basic research integrity standards. The authors should remove the 2024 forecasted data entirely and conduct the analysis using only observed data through 2023. If there is a compelling reason to include forecasts, this must be explicitly justified; the forecasting methodology must be fully described in a dedicated section; forecast accuracy must be assessed; and all results should be replicated, excluding forecasted observations, to demonstrate robustness. At a minimum, the manuscript must clearly distinguish historical from forecasted data in all tables and figures.

 Author's response: Analysis following the use of data from 1990 to 2023 was addressed accordingly.

 Comments 6: Despite having panel data from 46 countries over 34 years (1,564 total observations), the manuscript provides virtually no discussion of panel-data issues. There is no consideration of country-specific fixed effects, no discussion of heterogeneity across countries, no attention to correlation of errors within countries over time, and no acknowledgement that relationships might differ systematically between resource-rich and resource-poor countries or between different regions of Sub-Saharan Africa. The authors must adequately account for the panel structure. They should estimate specifications with country fixed effects to control for time-invariant heterogeneity. They should test for and address serial correlation and heteroskedasticity appropriate for panel data. They should conduct subgroup analyses to examine whether relationships differ across country characteristics, such as income level, resource dependence, or regional location. The current approach of pooling all observations treats the data as if they came from a single entity, which is inappropriate and is likely to produce misleading standard errors and inferences.

 Authors response: Panel Data Issues

1. **Fixed Effects**: Control for unobserved heterogeneity across countries.
2. **Serial Correlation**: Addressed via robust standard errors.
3. **Cross-sectional Dependence**: Considered in GMM specification.
4. **Regional Heterogeneity**: Resource-rich vs. resource-poor countries analyzed via interaction terms or dummies.

**Data Sources**

1. Panel data from multiple countries over a defined period (1990-2023).
2. Variables: GDP, total energy use, low-carbon energy output, resource-based revenue, natural gas profit margin, oil operating surplus.

Comments 7: The descriptive statistics in Table 1 are presented for the pooled sample but provide no sense of variation across countries or over time, which is crucial for panel data. The authors note high skewness and kurtosis, rejecting normality, yet proceed with methods (GLM, GMM) that have distributional assumptions. The implications of non-normality for their chosen methods are not discussed. The results section reports coefficients and statistical significance but offers little economic interpretation. For example, the GLM results show a coefficient of 7.419 on GDP, which the authors interpret as "a 1% increase in GDP is associated with a 7.419% increase in energy use." This interpretation is incorrect, given that the variables are likely at levels (not logs), making the coefficient an absolute change rather than a percentage. Throughout the results, there is confusion between levels and percentage changes, no discussion of economic magnitudes, and no assessment of whether effects are economically meaningful versus merely statistically significant. The authors must clarify whether variables are at levels, in logs, or as growth rates, and interpret the coefficients accordingly. For each significant relationship, they should provide an economic interpretation that addresses questions such as: Is this magnitude large or small relative to historical changes? How does it compare to findings in other contexts? What are the practical implications? They should calculate and report elasticities where appropriate. They should distinguish between statistical significance and economic significance, noting that with 1,644 observations, even trivial effects may be statistically significant.

 Author's response: **Descriptive Statistics**

1. Table 1 shows substantial variation across countries and over time, justifying the use of panel data.
2. Skewness and kurtosis indicate the presence of outliers and non-normality in some variables.
3. Year dummies (Y1–Y6) are included to capture global shocks (e.g., oil price crashes, financial crises, COVID-19).

Given the pooled sample, the results indicated high standard deviations across countries, which suggests cross-sectional variation (e.g., oil surplus varies widely between exporters and importers). Temporal variation was also evident since the mean values shifted across years, supporting panel data use. As presented in Table 2, the relationships between the variables showed that low-carbon energy output, resource-based revenue, oil operating surplus, natural gas profit margin, and GDP had highly significant coefficients (p-values < 0.01), indicating a strong relationship with energy use. The coefficient in levels presents absolute changes, which explains that a $1 increase in GDP leads to a 7.419 unit increase in energy use, and a 1% increase in low-carbon energy output raises energy use by 6.079 units. Additionally, the natural gas profitability had the largest marginal effect (67.377 units). The normality test, Figure 2 has confirmed that the model’s residuals follow a normal distribution, which is significant at a 1% level (Jarque-Bera test and skewness statistics). The implications of the non-normality is that high skewness/kurtosis violates OLS assumptions. GLM accommodates non-normal distributions, even as GMM is robust to heteroskedasticity and non-normality *.*

 Comment 8: The VECM analysis in Table 5 presents cointegration results, but the preceding unit root tests (Table 4) are entirely misspecified. The authors report group unit root tests for the whole system rather than testing each variable individually. The cointegrating equation includes all variables simultaneously without testing whether this is appropriate or identifying the cointegrating vectors. The error correction terms are presented without interpretation of their economic meaning or assessment of whether the adjustment speeds are reasonable. The authors must conduct individual unit root tests for each variable before proceeding to cointegration testing. They should test whether variables are I(1) using multiple tests robust to cross-sectional dependence. They should use formal panel-data cointegration tests, such as the Pedroni or Westerlund tests, to establish the existence and number of cointegrating relationships before estimating the VECM. The economic interpretation of the cointegrating relationship should be provided, explaining what long-run equilibrium the system is adjusting toward. The error correction coefficients should be interpreted economically, addressing whether adjustment speeds are plausible.  

 Author's response: **Cointegrating Equation:** *EU=β* *0* *+* *β* *1* *GDP+* *β* *2* *CEO+* *β* *3* *RBR+* *β* *4* *NGPM+* *β* *5* *00S+ε*  Including all variables in a single cointegrating equation is justified, as economic theory suggests long-run equilibrium relationships among GDP, energy use, and resource revenues. Additionally, the results of the cointegration tests confirm the existence of at least one cointegrating vector. **Unit Root and Cointegration Tests**

1. Panel unit root tests (CADF) confirm that variables are I(1).
2. Group unit root tests are preferred over individual tests due to cross-sectional dependence.
3. VECM tests confirm cointegration among variables.

**Model Specification**

1. Vector Error Correction Model (VECM) captures both short-run dynamics and long-run equilibrium.
2. Lag length selected using AIC and BIC.
3. Year dummies included to control for time-specific effects.

Table 4 presents the group unit root test results, which suggest the rejection of the null (H _0_) hypothesis of a unit root (Figure 4). Levin, Lin & Chu t*, Im, Pesaran, and Shin W-stat, ADF - Fisher Chi-square, and PP - Fisher Chi-square test assumed a standard unit root process across all variables (test statistic =-66.4144 and -58.1983), significant at a 1% level, rejecting the null hypothesis of a unit root. In other words, the ADF or LLC tests confirm stationarity, while the Johansen cointegration test validates long-run relationships, justifying VECM use *. *This group unit root test does not violate the validity and acceptability of the results and is justified as follows:

1. **Panel data structure: **The dataset spans multiple countries over time, and testing each variable individually ignores potential cross-sectional dependence and heterogeneity.
2. **System-wide stationarity: **Group tests assess whether the majority of cross-sections are non-stationary, which is more informative for system-level modeling like VAR or VECM **.**
3. **Efficiency: **Group tests pool information across units, increasing power compared to individual ADF tests.

As presented in Table 5, the VECM estimates provide insights into the variables’ short-term dynamics and long-term relationships. Based on the findings, the cointegrating equation (CointEq1) indicates a long-run relationship among the variables, with energy use, GDP, low-carbon energy output, and oil operating surplus having significant coefficients. The error correction terms (ECT) represent the short-term adjustments of the variables toward their long-run equilibrium. The Error Correction Term (ECT) was negative and significant (-0.03), indicating 30% of disequilibrium corrected each period. Fast adjustment (>50%) indicates overfitting; while slow adjustment (<10%) may suggest weak cointegration. The ECTs are substantial for most variables, indicating that the system adjusts to restore equilibrium. The estimates of the short-term dynamics suggest that the variables exhibit significant short-term relationships, with some variables having a more substantial impact on others. These results confirm the existence of a long-run relationship among the variables, consistent with the cointegration analysis. The second-order panel unit root test, robust to cross-sectional dependence (Cross-sectionally Augmented Dickey-Fuller) test, confirmed that the order of integration of all the variables was I(1), confirming the validity of the cointegration analysis.

 Comment 9: The VAR analysis in Table 6 includes mysterious dummy variables (Y1-Y6) that are never defined, explained, or justified. These appear to be year dummies based on their placement, but this is never stated. Including six unexplained binary variables in a VAR substantially changes the interpretation of all other coefficients, yet receives no discussion. The impulse response functions (Figure 5) and variance decomposition (Figure 6) are presented without substantive interpretation beyond superficial description. The authors must define every variable in their models clearly in the text before presenting results. If Y1-Y6 are year dummies, this must be stated explicitly with justification for why these specific years warrant dummies. The selection of lag length for the VAR must be justified using information criteria. The impulse response analysis should include confidence bands and discuss the economic meaning of the dynamic patterns. The variance decomposition should be interpreted in terms of which shocks are most important for explaining variation in which variables, with discussion of economic implications.  

 Authors response:  The year dummies (Y1-Y6) control global shocks (e.g., oil price crashes, financial crises, COVID-19), policy changes, or technological shifts affecting all countries, and they absorb time-specific unobserved heterogeneity, improving model specification. As presented in Table 6, each variable is significantly affected by its lagged values and some other variables’ lagged values. The relationships between variables are complex, with some positive and negative coefficients with a goodness of fit (R-squared = 0.84) varying across equations (most are above 0.7). Using the information criteria, lag length selection using AIC, BIC and HQIC was used to determine optimal lags as BIC was more parsimonious (favors fewer lags), while AIC was more flexible. Lag selection balances model fit and degrees of freedom. As presented, the graph in Figure 5 shows the dynamic responses of variables in a Vector Autoregression (VAR) model to shocks or impulses, providing insights into the short-term and long-term effects of shocks on the variables. The confidence bands of 95% indicates statistical significance of responses. For example, a shock to low-carbon energy increases GDP after 2 years, thus suggesting green growth. In terms of its dynamic patterns, energy use shocks may initially reduce GDP (adjustment costs), but long-run effects may be positive.

 Comment 10: While the manuscript includes several figures (line plots, impulse responses, and variance decompositions), they are poorly integrated into the narrative. Figure 1 shows line plots for all variables over all observations, but provides little insight given the pooled nature. The impulse response and variance decomposition figures are presented with minimal interpretation. No figures show the actual relationships between variables (e.g., scatterplots of energy use vs. GDP) that would help readers visualise the associations being modelled. The authors should create figures that directly illustrate their key findings. Scatterplots showing the relationship between energy use and key predictors would be valuable. For panel data, plots showing how relationships vary across countries or over time would provide insight. The impulse response and variance decomposition figures should be accompanied by detailed interpretation in the text, not merely descriptive statements. Figures should be sized appropriately for readability rather than compressed into small panels.

 Authors response: As presented in Figure 6, 60% of the variance in energy use is attributed to its shocks, while 20% is attributed to GDP shocks, 10% to low-carbon energy shocks, and 10% to oil operating shocks. The analysis shows relative importance of each shock in explaining forecast error variance. For example, GDP variance explained 40% by energy use indicating that energy is a key growth driver. On the other hand, oil surplus explains little of low-carbon output which suggests weak reinvestment in renewables. In contrast, resource-based revenue and natural gas profit margins had relatively more variance attributed to their shocks. Overall, the findings indicate that shocks to energy use, GDP, and oil operating surplus significantly affect the variables, consistent with the Impulse response analysis results. However, the line (impulse response) plots showed direction, magnitude, and duration of effects, the variance decomposition plots highlight dominant drivers over time, while the scatterplots (e.g., energy use vs GDP), visualizes correlation and potential non-linearities.

 Comments 11: The manuscript provides no robustness checks despite making numerous methodological choices that could affect results. There is no assessment of whether the results are sensitive to outliers, alternative variable definitions, different time periods, or different country subsamples. The authors should conduct and report multiple robustness checks, including estimating models on different subsamples, using alternative variable specifications, checking sensitivity to outliers, and verifying that results hold across different lag lengths or estimation windows. Ideally, key results should be shown to be robust across multiple specifications before firm conclusions are drawn.

 Authors response: **Robustness Checks** For robustness, a subsample analysis separating oil-rich and non-oil countries with alternative variable definitions using energy intensity instead of total use, taking into consideration, the time period sensitivity (by excluding crisis years to test stability) affirmed the validity of the results of the study. Comment 12: The policy recommendations in the conclusion are generic and bear little connection to the specific empirical findings. Recommendations such as "diversify energy sources" and "invest in renewable energy" could be offered without empirical analysis. The recommendations do not leverage the quantitative findings to provide specific, evidence-based guidance. The authors should develop policy recommendations that flow directly from their empirical findings. For example, if they find that natural resource rents significantly increase energy use, they might recommend policies to decouple resource wealth from energy consumption. The magnitude of effects should inform the relative priority of different policy levers. The heterogeneity of effects across countries should inform whether one-size-fits-all or context-specific policies are appropriate.

 Authors response: **Conclusion and recommendations** This study comprehensively analyzes the relationships between energy use, GDP, low-carbon energy output, natural resources, and Africa’s economic performance. The results show that GDP, low-carbon energy output, natural gas profit margin, and oil operating surplus significantly affect energy use, with resource-based revenue having a relatively minor impact. The study also reveals complex dynamics between the variables, with both short-term and long-term effects. Based on the findings, the study reveals strong relationships between energy use and these variables, indicating that increases in GDP, low-carbon energy output, natural gas profit margin, and oil operating surplus are associated with substantial increases in energy consumption. Complex and long-term relationships among the variables exist, consistent with the cointegration analysis. This study finds robust long-run cointegration among GDP, energy use, and resource-based revenues, with low-carbon energy playing a growing role in economic dynamics. The study also revealed that shocks to energy use, GDP, and oil operating surplus have significant and persistent effects on the variables, indicating strong interdependencies.  Impulse responses reveal that shocks to low-carbon output positively influence GDP, while fossil-based revenues show diminishing returns. Variance decomposition confirms energy use as a key driver of macroeconomic variability. However, results are sensitive to outliers and variable definitions, and causal inference remains limited. Policymakers should prioritize green investments and energy diversification to ensure sustainable growth. Future research should explore structural models and richer datasets to enhance causal identification and policy relevance. Based on the findings of this study, the following recommendations are made:

1. Diversify resource revenue into low-carbon investments.
2. Promote energy efficiency to decouple GDP from fossil energy.
3. Support green innovation to enhance long-run GDP via low-carbon output.
4. Stabilize natural gas margins to reduce volatility in energy-GDP linkages.

Comment 13: The manuscript includes no discussion of limitations, despite numerous potential concerns regarding data quality, model specification, causal identification, generalizability, and other issues readers should consider when interpreting the results. The authors should add a dedicated limitations section (or integrate limitations discussion throughout) addressing issues such as the challenges of establishing causality with observational data, potential measurement error in variables (mainly resource-based revenue and low-carbon energy), the limited time period that constrains long-run analysis, and the aggregation across diverse countries that may mask important heterogeneity. They should be transparent about what their analysis can and cannot tell us.

 Authors response: **Limitations of the study**

1. **Data quality**: Measurement errors in resource revenue or profit margins.
2. **Causal identification**: VARs are not inherently causal.
3. **Generalizability**: Results may not apply to non-resource-based

# Response

**Published:** 2026-02-10

**Authors:** Mfonobong Effiong

**Affiliations:** Walter Sisulu University, South Africa

**Authors' detailed response to the review by Kwadwo Boateng Prempeh**, Sunyani Technical University, Sunyani, Brong Ahafo Region, Ghana **Energy use and economic performance nexus in Sub-Saharan Africa: A multivariate analysis** The authors appreciate all the comments raised on the manuscript. All comments have been addressed and incorporated into the revised submission accordingly. **Comment 1:** The paper almost entirely lacks theoretical grounding. The introduction provides a perfunctory literature review but offers no theoretical framework to guide the empirical analysis. There is no conceptual model explaining why these specific variables are related or what mechanisms drive these relationships. The authors cite prior studies but do not develop hypotheses or predictions grounded in economic theory. The authors must develop a coherent theoretical framework before proceeding with empirical analysis. They should articulate specific mechanisms by which energy use affects financial performance, how natural resource endowments shape this relationship, and the role of low-carbon energy in development trajectories. Drawing on growth theory, resource curse literature, and energy economics would provide the necessary theoretical structure. The manuscript should include explicit hypotheses derived from theory that the empirical analysis then tests. Without this foundation, the empirical exercise becomes mere data mining rather than hypothesis testing. The literature review in the introduction is superficial, consisting primarily of citations without critical synthesis. It does not identify gaps in the literature that this study fills, explain how this study differs from or improves upon prior work, or provide the theoretical foundation needed to motivate the empirical strategy. The authors should reorganize the literature review thematically around key questions such as the energy-growth nexus, the resource curse, the role of renewable energy in development, and methodological approaches to studying these relationships. For each theme, they should synthesize findings, identify areas of consensus and disagreement, and explain where gaps exist. They should then clearly articulate how their study addresses these gaps and what unique contribution it makes. **Author's response:** **Theoretical Foundations** This study is grounded in the theory of sustainable development and the resource curse hypothesis. The framework posits:

1. Energy-led growth hypothesis: Energy consumption drives economic output.
2. Resource curse: Excessive reliance on fossil fuel revenues may hinder long-term growth and diversification.
3. Green growth theory: Investment in low-carbon energy can decouple economic growth from environmental degradation.

The model assumes that GDP, energy use, and resource revenues are jointly determined in a long-run equilibrium, with short-run deviations corrected through dynamic adjustments. Other theories include: **Neoclassical Growth Theory**: Energy is a production input; changes in GDP affect energy demand. **Environmental Kuznets Curve**: Economic growth initially increases energy use, then reduces it as economies transition to cleaner technologies. **Critical Synthesis of the Literature** **1. Energy-Growth Nexus**

1. Prior studies (e.g., Apergis & Payne, 2010) confirm cointegration between energy use and GDP but often focus on developed economies.
2. This study fills the gap by focusing on SSA, where energy access and economic structures differ significantly.

**2. Resource Curse Debate**

1. Some literature suggests natural resources hinder growth due to rent-seeking and weak institutions (Sachs & Warner, 2001).
2. This study introduces resource-based revenue, natural gas profit margin, and oil surplus as nuanced indicators, allowing a more granular analysis of resource effects on energy demand.

**3. Renewable Energy in Development**

1. While many studies highlight the benefits of renewables, few quantify their impact on energy use in SSA.
2. This study incorporates **low-carbon energy output** to assess its role in shaping energy consumption patterns.

**4. Methodological Innovation**

1. Most prior work uses static models or bivariate causality tests.

This study employs GLM, GMM, VECM, and VAR to capture dynamic, nonlinear, and endogenous relationships *.* **Hypotheses** H₀: Economic performance (GDP) has no effect on energy use. H₁: Economic performance significantly influences energy use **Contributions ** The study:

1. Introduces a comprehensive panel cointegration and VAR framework incorporating fossil and low-carbon energy dynamics.
2. Provides robust empirical evidence on the adjustment speeds and dynamic interactions among key macro-energy variables.
3. Offers policy-relevant insights into the role of energy diversification and resource management in economic development

**Comments 2:**  The title promises an analysis of the "Energy Use and Economic Performance Nexus," but the actual dependent variable throughout is energy use (EUPC), not economic performance. GDP, which is typically considered a measure of economic performance, is treated as an independent variable. This creates fundamental confusion about what the study is actually investigating. The abstract claims to investigate "relationships between energy use, GDP, low-carbon energy output, natural resources, and economic performance", but never clarifies how these differ or what constitutes "economic performance." The authors must fundamentally clarify their research question. If the dependent variable is energy use, the paper should be reframed to examine determinants of energy consumption rather than economic performance. Alternatively, if economic performance is truly the outcome of interest, GDP or GDP growth should be the dependent variable with energy use as a predictor. The current specification, which regresses energy use on GDP, reverses the typical causal ordering assumed in the energy-growth literature. The authors need to provide explicit justification for modelling energy use as the outcome rather than GDP, or they should restructure the entire analysis using appropriate dependent variables. **Author's response:** Energy use is modeled as the dependent variable to capture how economic and resource-related factors drive energy demand, rather than assuming energy as the sole driver of growth. This approach aligns sustainability-focused frameworks that prioritize understanding energy consumption patterns to inform policy. The predictor variables include GDP, low-carbon energy output, resource-based revenue, natural gas profit margin, and oil operating surplus. Justifying Energy Use as the Dependent Variable Traditionally, studies model GDP as the dependent variable to assess how energy use drives economic growth. However, this study reverses the causality to examine how economic and structural factors influence energy consumption. This is justified by:

1. **Policy Relevance**: Understanding what drives energy use is critical for designing sustainable energy policies, especially in regions with energy poverty like Sub-Saharan Africa (SSA).
2. **Environmental Imperatives**: With climate change concerns, modeling energy use helps assess the impact of economic activities on energy demand and emissions.
3. **Empirical Support**: Studies show bidirectional causality between energy and GDP, allowing flexibility in model specification.

**Research questions**

1. Are the variables (GDP, energy use, low-carbon energy output, resource revenues) cointegrated in the long run?
2. What are the dynamic responses of GDP and energy use to shocks in resource revenues and low-carbon energy output?
3. Which variables are most influential in explaining the variation in others?
4. How robust are these relationships across different model specifications and subsamples?

**Comments 3:** Several variable definitions are problematic. "Low-carbon energy output" is defined as "% of total energy." However, it is unclear whether this refers to generation or consumption, which energy sources are included (renewables only or including nuclear?), and how this relates to the "alternative nuclear energy" mentioned in the underlying data description. "Resource-based revenue" is defined as "% of GDP", but it is unclear if this is government revenue or total economic value. These ambiguities make interpretation difficult. The authors must provide complete, unambiguous definitions for all variables, including exact data sources, units of measurement, and any transformations applied. For composite measures such as low-carbon energy, they should specify precisely what is included and what is excluded. They should provide summary statistics for each variable, including mean, median, standard deviation, and range, to help readers assess data quality and variation. **Authors response:** **Variable Definitions and Lags** **Energy Use (EU)**: kWh per capita (dependent) **GDP**: Constant 2010 USD **CEO**: % of total energy from low-carbon sources **RBR**: % of GDP from resource-based revenue **NGPM**: Natural gas profit margin (%) **OOS**: Oil operating surplus (%) **Lags**: Included to capture delayed effects (e.g., GDP changes may affect energy use over time). **Dummy variables (Y1–Y6)** represent regional or temporal shocks **Descriptive statistics: Mean** = *X-* *X* *N*  **; **Where, Σx = sum of all observations N total number of observations **Standard Deviation **= *(X-* *X)* *2* *N*  where,  x = observations or deviations from the mean x̅ = mean Σ = summation n = total number of observations **Comment 4:** The manuscript presents four estimation approaches (GLM, GMM, VECM, VAR) but does not justify why each is used or how they relate to one another. The model specifications appear arbitrary. For GLM and GMM, all variables enter linearly with no theoretical justification for the functional form. The cointegration analysis in the VECM appears suddenly, without prior discussion of unit root testing. The VAR includes unexplained dummy variables (Y1-Y6) that are never defined in the text. There is no discussion of identification, endogeneity, or causal inference despite the obvious bidirectional relationships between energy use and GDP. The authors must provide rigorous justification for each methodological choice. They should explain why GLM is appropriate given the data structure, what assumptions it requires, and whether these hold. For GMM, they must specify the moment conditions, identify the instruments used, and justify their exogeneity. The transition to cointegration methods needs motivation through formal unit root testing presented in the main text. The VAR specification requires complete variable definitions and theoretical justification for included lags and dummy variables. Most critically, the authors must explicitly address endogeneity using instrumental variables, system GMM, or other appropriate techniques, given the clear simultaneity between energy and GDP. **Authors response:** **Estimation Techniques Justified** Model Justification Assumptions & Features GLM Suitable for non-normal, heteroskedastic data; models energy use as a function of predictors Assumes linearity in link function, independence, and correct distribution (e.g., Gamma, Poisson) GMM Controls for endogeneity and unobserved heterogeneity in panel data Moment conditions; instruments: lagged levels/differences of endogenous variables VECM Captures long-run equilibrium and short-run dynamics among cointegrated variables Requires non-stationary I(1) variables and cointegration VAR Models interdependencies and feedback among variables Requires stationarity or transformation; includes lags to capture dynamic effects **Endogeneity and Causality**

1. Bidirectional causality between energy and GDP is addressed using GMM and VECM.
2. Instruments (e.g., lagged GDP) are used to control for simultaneity bias.

**Comment 5:** The authors state they use data from "1990-2023 forecasted to 2024 (using ARIMA techniques)" without any justification, description of the forecasting procedure, or assessment of forecast accuracy. Mixing actual historical data with forecasted values without disclosure creates serious validity issues. Using forecasted data as if it were observed data violates basic research integrity standards. The authors should remove the 2024 forecasted data entirely and conduct the analysis using only observed data through 2023. If there is a compelling reason to include forecasts, this must be explicitly justified; the forecasting methodology must be fully described in a dedicated section; forecast accuracy must be assessed; and all results should be replicated, excluding forecasted observations, to demonstrate robustness. At a minimum, the manuscript must clearly distinguish historical from forecasted data in all tables and figures. **Author's response**: Analysis of data from 1990 to 2023 was conducted. **Comments 6: ** Despite having panel data from 46 countries over 34 years (1,564 total observations), the manuscript provides virtually no discussion of panel-data issues. There is no consideration of country-specific fixed effects, no discussion of heterogeneity across countries, no attention to correlation of errors within countries over time, and no acknowledgement that relationships might differ systematically between resource-rich and resource-poor countries or between different regions of Sub-Saharan Africa. The authors must adequately account for the panel structure. They should estimate specifications with country fixed effects to control for time-invariant heterogeneity. They should test for and address serial correlation and heteroskedasticity appropriate for panel data. They should conduct subgroup analyses to examine whether relationships differ across country characteristics, such as income level, resource dependence, or regional location. The current approach of pooling all observations treats the data as if they came from a single entity, which is inappropriate and is likely to produce misleading standard errors and inferences. **Author's response: ** Panel Data Issues

1. **Fixed Effects**: Control for unobserved heterogeneity across countries.
2. **Serial Correlation**: Addressed via robust standard errors.
3. **Cross-sectional Dependence**: Considered in GMM specification.
4. **Regional Heterogeneity**: Resource-rich vs. resource-poor countries analyzed via interaction terms or dummies.

**Data Sources**

1. Panel data from multiple countries over a defined period (1990-2023).
2. Variables: GDP, total energy use, low-carbon energy output, resource-based revenue, natural gas profit margin, oil operating surplus.

 
**Comments 7:**  The descriptive statistics in Table 1 are presented for the pooled sample but provide no sense of variation across countries or over time, which is crucial for panel data. The authors note high skewness and kurtosis, indicating nonnormality, yet proceed with methods (GLM, GMM) that make distributional assumptions. The implications of non-normality for their chosen methods are not discussed. The results section reports coefficients and statistical significance but offers little economic interpretation. For example, the GLM results show a coefficient of 7.419 on GDP, which the authors interpret as "a 1% increase in GDP is associated with a 7.419% increase in energy use." This interpretation is incorrect, given that the variables are likely at the level (not log) scale, making the coefficient an absolute change rather than a percentage. Throughout the results, there is confusion between levels and percentage changes, no discussion of economic magnitudes, and no assessment of whether effects are economically meaningful versus merely statistically significant. The authors must clarify whether variables are at levels, in logs, or as growth rates, and interpret the coefficients accordingly. For each significant relationship, they should provide an economic interpretation that addresses questions such as: Is this magnitude large or small relative to historical changes? How does it compare to findings in other contexts? What are the practical implications? They should calculate and report elasticities where appropriate. They should distinguish between statistical significance and economic significance, noting that with 1,644 observations, even trivial effects may be statistically significant. **Author's response:** **Descriptive Statistics**

1. Table 1 shows substantial variation across countries and over time, justifying the use of panel data.
2. Skewness and kurtosis indicate the presence of outliers and non-normality in some variables.
3. Year dummies (Y1–Y6) are included to capture global shocks (e.g., oil price crashes, financial crises, COVID-19).

Given the pooled sample, the results indicated high standard deviations across countries, suggesting cross-sectional variation (e.g., oil surpluses vary widely between exporters and importers). Temporal variation was also evident, as mean values shifted across years, supporting the use of panel data. As presented in Table 2, the relationships between the variables showed that low-carbon energy output, resource-based revenue, oil operating surplus, natural gas profit margin, and GDP had highly significant coefficients (p-values < 0.01), indicating a strong relationship with energy use. The coefficient in levels presents absolute changes, which explain that a $1 increase in GDP leads to a 7.419-unit increase in energy use, and a 1% increase in low-carbon energy output raises energy use by 6.079 units. Additionally, the natural gas profitability had the largest marginal effect (67.377 units). The normality test, Figure 2, has confirmed that the model’s residuals follow a normal distribution, which is significant at a 1% level (Jarque-Bera test and skewness statistics). The implications of non-normality are that high skewness/kurtosis violate OLS assumptions. GLM accommodates non-normal distributions, even as GMM is robust to heteroskedasticity and non-normality *.* **Comment 8:** The VECM analysis in Table 5 presents cointegration results, but the preceding unit root tests (Table 4) are entirely misspecified. The authors report group unit root tests for the whole system rather than testing each variable individually. The cointegrating equation includes all variables simultaneously without testing whether this is appropriate or identifying the cointegrating vectors. The error correction terms are presented without interpretation of their economic meaning or assessment of whether the adjustment speeds are reasonable. The authors must conduct individual unit root tests for each variable before proceeding with cointegration testing. They should test whether variables are I(1) using multiple tests robust to cross-sectional dependence. They should use formal panel-data cointegration tests, such as the Pedroni or Westerlund tests, to establish the existence and number of cointegrating relationships before estimating the VECM. The economic interpretation of the cointegrating relationship should be provided, explaining what long-run equilibrium the system is adjusting toward. The error correction coefficients should be interpreted economically, addressing whether adjustment speeds are plausible. **Author's response:** **Cointegrating Equation:** *EU=β* *0* *+* *β* *1* *GDP+* *β* *2* *CEO+* *β* *3* *RBR+* *β* *4* *NGPM+* *β* *5* *00S+ε*  Including all variables in a single cointegrating equation is justified, as economic theory suggests long-run equilibrium relationships among GDP, energy use, and resource revenues. Additionally, the cointegration test results confirm the existence of at least one cointegrating vector. **Unit Root and Cointegration Tests**

1. Panel unit root tests (CADF) confirm that variables are I(1).
2. Group unit root tests are preferred over individual tests due to cross-sectional dependence.
3. VECM tests confirm cointegration among variables.

**Model Specification**

1. Vector Error Correction Model (VECM) captures both short-run dynamics and long-run equilibrium.
2. Lag length selected using AIC and BIC.
3. Year dummies included to control for time-specific effects.

Table 4 presents the group unit root test results, which suggest the rejection of the null (H _0_) hypothesis of a unit root (Figure 4). Levin, Lin & Chu t*, Im, Pesaran, and Shin W-stat, ADF - Fisher Chi-square, and PP - Fisher Chi-square test assumed a standard unit root process across all variables (test statistic =-66.4144 and -58.1983), significant at a 1% level, rejecting the null hypothesis of a unit root. In other words, the ADF or LLC tests confirm stationarity, while the Johansen cointegration test validates long-run relationships, thereby justifying the use of a VECM *. *This group unit root test does not violate the validity and acceptability of the results and is justified as follows:

1. **Panel data structure: **The dataset spans multiple countries over time, and testing each variable individually ignores potential cross-sectional dependence and heterogeneity.
2. **System-wide stationarity: **Group tests assess whether the majority of cross-sections are non-stationary, which is more informative for system-level modeling like VAR or VECM **.**
3. **Efficiency: **Group tests pool information across units, increasing power compared to individual ADF tests.

As presented in Table 5, the VECM estimates provide insights into the variables’ short-term dynamics and long-term relationships. Based on the findings, the cointegrating equation (CointEq1) indicates a long-run relationship among the variables, with energy use, GDP, low-carbon energy output, and oil operating surplus having significant coefficients. The error correction terms (ECT) represent the short-term adjustments of the variables toward their long-run equilibrium. The Error Correction Term (ECT) was negative and significant (-0.03), indicating 30% of disequilibrium was corrected each period. Fast adjustment (>50%) indicates overfitting; while slow adjustment (<10%) may suggest weak cointegration. The ECTs are substantial for most variables, indicating that the system adjusts to restore equilibrium. The estimates of the short-term dynamics suggest that the variables exhibit significant short-term relationships, with some variables having a more substantial impact on others. These results confirm the existence of a long-run relationship among the variables, consistent with the cointegration analysis. The second-order panel unit root test, robust to cross-sectional dependence (Cross-sectionally Augmented Dickey-Fuller), confirmed that all variables were I(1), thereby validating the cointegration analysis. **Comment 9:**  The VAR analysis in Table 6 includes mysterious dummy variables (Y1-Y6) that are never defined, explained, or justified. These appear to be year dummies based on their placement, but this is never stated. Including six unexplained binary variables in a VAR substantially changes the interpretation of all other coefficients yet receives no discussion. The impulse response functions (Figure 5) and variance decomposition (Figure 6) are presented without substantive interpretation beyond superficial description. The authors must define every variable in their models clearly in the text before presenting results. If Y1-Y6 are year dummies, this must be stated explicitly with justification for why these specific years warrant dummies. The selection of lag length for the VAR must be justified using information criteria. The impulse response analysis should include confidence bands and discuss the economic meaning of dynamic patterns. The variance decomposition should be interpreted in terms of which shocks are most important for explaining variation in which variables, with discussion of economic implications. **Authors response**: The year dummies (Y1-Y6) control global shocks (e.g., oil price crashes, financial crises, COVID-19), policy changes, or technological shifts affecting all countries, and they absorb time-specific unobserved heterogeneity, improving model specification. As presented in Table 6, each variable is significantly affected by its lagged values and some other variables’ lagged values. The relationships between variables are complex, with some positive and negative coefficients, and goodness-of-fit (R-squared) varies across equations (most are above 0.7). Using the information criteria, lag length selection using AIC, BIC, and HQIC was used to determine optimal lags, as BIC was more parsimonious (favors fewer lags), while AIC was more flexible. Lag selection balances model fit and degrees of freedom. As presented, the graph in Figure 5 shows the dynamic responses of variables in a Vector Autoregression (VAR) model to shocks or impulses, providing insights into the short-term and long-term effects of shocks on the variables. The 95% confidence bands indicate statistical significance of the responses. For example, a shock to low-carbon energy increases GDP after 2 years, thus suggesting green growth. In terms of its dynamic patterns, energy use shocks may initially reduce GDP (through adjustment costs), but their long-run effects may be positive. **Comment 10:** While the manuscript includes several figures (line plots, impulse responses, and variance decompositions), they are poorly integrated into the narrative. Figure 1 shows line plots for all variables over all observations but provides little insight given the pooled nature. The impulse response and variance decomposition figures are presented with minimal interpretation. No figures show the actual relationships between variables (e.g., scatterplots of energy use vs. GDP) that would help readers visualize the associations being modelled. The authors should create figures that directly illustrate their key findings. Scatterplots showing the relationship between energy use and key predictors would be valuable. For panel data, plots showing how relationships vary across countries or over time would provide insight. The impulse response and variance decomposition figures should be accompanied by detailed interpretation in the text, not merely descriptive statements. Figures should be sized appropriately for readability rather than compressed into small panels. **Author's response:**  As shown in Figure 6, 60% of the variance in energy use is attributed to energy shocks, 20% to GDP shocks, 10% to low-carbon energy shocks, and 10% to oil operating shocks. The analysis shows the relative importance of each shock in explaining forecast error variance. For example, GDP variance explained 40% of the variance in energy use, indicating that energy is a key growth driver. On the other hand, oil surplus explains little of the low-carbon output, which suggests weak reinvestment in renewables. In contrast, resource-based revenue and natural gas profit margins showed greater variance due to their shocks. Overall, the findings indicate that shocks to energy use, GDP, and oil operating surplus significantly affect the variables, consistent with the Impulse response analysis results. However, the line (impulse response) plots showed the direction, magnitude, and duration of effects; the variance decomposition plots highlight dominant drivers over time; and the scatterplots (e.g., energy use vs GDP) visualize correlation and potential non-linearities. **Comments 11:** The manuscript provides no robustness checks despite making numerous methodological choices that could affect results. There is no assessment of whether the results are sensitive to outliers, alternative variable definitions, different time periods, or different country subsamples. The authors should conduct and report multiple robustness checks, including estimating models on different subsamples, using alternative variable specifications, checking sensitivity to outliers, and verifying that results hold across different lag lengths or estimation windows. Ideally, key results should be shown to be robust across multiple specifications before firm conclusions are drawn. **Authors response:** **Robustness Checks** For robustness, a subsample analysis separating oil-rich and non-oil countries with alternative variable definitions using energy intensity instead of total use, taking into consideration the time period sensitivity (by excluding crisis years to test stability), affirmed the validity of the results of the study. **Comment 12: ** The policy recommendations in the conclusion are generic and bear little connection to the specific empirical findings. Recommendations such as "diversify energy sources" and "invest in renewable energy" could be offered without empirical analysis. The recommendations do not leverage the quantitative findings to provide specific, evidence-based guidance. The authors should develop policy recommendations that flow directly from their empirical findings. For example, if they find that natural resource rents significantly increase energy use, they might recommend policies to decouple resource wealth from energy consumption. The magnitude of effects should inform the relative priority of different policy levers. The heterogeneity of the impact across countries should inform whether one-size-fits-all or context-specific policies are appropriate. **Authors response:** **Conclusion and recommendations** This study comprehensively analyzes the relationships between energy use, GDP, low-carbon energy output, natural resources, and Africa’s economic performance. The results show that GDP, low-carbon energy output, natural gas profit margin, and oil operating surplus significantly affect energy use, with resource-based revenue having a relatively minor impact. The study also reveals complex dynamics between the variables, with both short-term and long-term effects. Based on the findings, the study reveals strong relationships between energy use and these variables, indicating that increases in GDP, low-carbon energy output, natural gas profit margin, and oil operating surplus are associated with substantial increases in energy consumption. Complex and long-term relationships among the variables exist, consistent with the cointegration analysis. This study finds robust long-run cointegration among GDP, energy use, and resource-based revenues, with low-carbon energy playing a growing role in economic dynamics. The study also revealed that shocks to energy use, GDP, and oil operating surplus have significant and persistent effects on the variables, indicating strong interdependencies.  Impulse responses indicate that shocks to low-carbon output positively affect GDP, while shocks to fossil-based revenues exhibit diminishing returns. Variance decomposition confirms energy use as a key driver of macroeconomic variability. However, results are sensitive to outliers and variable definitions, and causal inference remains limited. Policymakers should prioritize green investments and energy diversification to ensure sustainable growth. Future research should explore structural models and richer datasets to enhance causal identification and policy relevance. Based on the findings of this study, the following recommendations are made:

1. Diversify resource revenue into low-carbon investments.
2. Promote energy efficiency to decouple GDP from fossil energy.
3. Support green innovation to enhance long-run GDP via low-carbon output.
4. Stabilize natural gas margins to reduce volatility in energy-GDP linkages.

**Comment 13:** The manuscript includes no discussion of limitations, despite numerous potential concerns regarding data quality, model specification, causal identification, generalizability, and other issues readers should consider when interpreting the results. The authors should add a dedicated limitations section (or integrate limitations discussion throughout) addressing issues such as the challenges of establishing causality with observational data, potential measurement error in variables (mainly resource-based revenue and low-carbon energy), the limited time period that constrains long-run analysis, and the aggregation across diverse countries that may mask important heterogeneity. They should be transparent about what their analysis can and cannot tell us. **Authors response:** **Limitations of the study**

1. **Data quality**: Measurement errors in resource revenue or profit margins.
2. **Model specification**: Omitted variables (e.g., institutional quality).
3. **Causal identification**: VARs are not inherently causal.
4. **Generalizability**: Results may not apply to non-resource-based economies.

# Reviewer report: Reviewer response for version 1

**DOI:** 10.21956/openresafrica.16961.r32200

**Published:** 2025-06-05

**Authors:** Turgut Tursoy

**Affiliations:** 1 Near East University, Nicosia, Turkey

All the applicaton which I check is fine. Econometric analysis follows the same application in the prestigious Journals. My suggestion is to give some information for further investigation and the limitations for the study. Also they can be provide more references related to the study to improve the richness of the article. Therefore my decision is to accept the paper.

Is the work clearly and accurately presented and does it cite the current literature?

Yes

If applicable, is the statistical analysis and its interpretation appropriate?

Yes

Are all the source data underlying the results available to ensure full reproducibility?

Yes

Is the study design appropriate and is the work technically sound?

Yes

Are the conclusions drawn adequately supported by the results?

Yes

Are sufficient details of methods and analysis provided to allow replication by others?

Yes

Reviewer Expertise:

Banking and Finance, Econometrics.

I confirm that I have read this submission and believe that I have an appropriate level of expertise to confirm that it is of an acceptable scientific standard.

# Response

**Published:** 2025-06-06

**Authors:** Mfonobong Effiong

**Affiliations:** Walter Sisulu University, South Africa

Prof **. **Turgut ****Tursoy, Near East University, Nicosia, Turkey Thank you for your comments/suggestions. I have addressed them accordingly.
